# Trehalose polyphleates participate in *Mycobacterium abscessus* fitness and pathogenesis

**DOI:** 10.1128/mbio.02970-24

**Published:** 2024-10-30

**Authors:** Silke Malmsheimer, Wassim Daher, Yara Tasrini, Claire Hamela, John Jairo Aguilera-Correa, Christian Chalut, Graham F. Hatfull, Laurent Kremer

**Affiliations:** 1 Centre National de la Recherche Scientifique UMR 9004, Institut de Recherche en Infectiologie de Montpellier (IRIM), Université de Montpellier, Montpellier, France; 2 INSERM, IRIM, Montpellier, France; 3 Institut de Pharmacologie et de Biologie Structurale (IPBS), Université de Toulouse, CNRS, Université Toulouse III – Paul Sabatier (UT3), Toulouse, France; 4 Department of Biological Sciences, University of Pittsburgh, Pittsburgh, Pennsylvania, USA; UCLA School of Medicine, Los Angeles, California, USA

**Keywords:** *Mycobacterium abscessus*, trehalose polyphleates, MmpL10, infection, pathogenesis, macrophage, zebrafish

## Abstract

Mycobacteria produce a large repertoire of surface-exposed lipids with major biological functions. Among these lipids, trehalose polyphleates (TPPs) are instrumental in the infection of *Mycobacterium abscessus* by the therapeutic phage BPs. However, while the biosynthesis and transport of TPPs across the membrane by MmpL10 have been reported, the role of TPPs in host infection remains enigmatic. Here, we addressed whether the loss of TPPs influences interactions with macrophages and the virulence of *M. abscessus*. As anticipated, the deletion of *mmpL10* in smooth (S) and rough (R) variants of *M. abscessus* abrogated TPP production, which was rescued upon gene complementation. Importantly, infection of human THP-1 cells with the *mmpL10* mutants was associated with decreased intramacrophage survival and a reduced proportion of infected cells. The rough *mmpL10* mutant showed an impaired capacity to block phagosomal acidification and was unable to co-localize with Galectin-3, a marker of phagosomal membrane damage. This suggests that TPPs participate, directly or indirectly, in phagolysosomal fusion and in phagosomal membrane damage to establish cytosolic communication. The TPP defect that affects the fitness and virulence of *M. abscessus* was further demonstrated in zebrafish embryos using a rough clinical strain resistant to phage BPs and harboring a frameshift mutation in *mmpL10*. Infection with this strain was correlated with a slight decrease in embryo survival and a reduced bacterial burden as compared to the corresponding parental and complemented derivatives. Together, these results indicate that TPPs are important surface lipids contributing to the pathogenicity of *M. abscessus*.

**IMPORTANCE:**

Trehalose polyphleates (TPPs) are complex lipids associated with the mycobacterial cell surface and were identified 50 years ago. While the TPP biosynthetic pathway has been described recently, the role of these lipids in the biology of mycobacteria remains yet to be established. The wide distribution of TPPs across mycobacterial species suggests that they may exhibit important functions in these actinobacteria. Here, we demonstrate that *Mycobacterium abscessus,* an emerging multidrug-resistant pathogen that causes severe lung diseases in cystic fibrosis patients, requires TPPs for survival in macrophages and virulence in a zebrafish model of infection. These findings support the importance of this underexplored family of lipids in mycobacterial pathogenesis.

## INTRODUCTION

*Mycobacterium abscessus* is a rapidly-growing non-tuberculous mycobacterium (NTM) of increasing clinical concern (1). Emerging as an opportunistic pathogen, it causes a range of infections in humans, most commonly affecting the skin and subjacent soft tissues, but it can also cause severe lung infections in immunocompromised patients or patients with cystic fibrosis or chronic obstructive pulmonary diseases (1, 2). *M. abscessus* can form biofilms, structured bacterial communities embedded within a self-produced polymeric matrix, where complex and diverse microbial interactions govern their behavior. These biofilms are observed both in environmental settings (3, 4) and during infection of pulmonary epithelial tissue (5) or on medical devices (6, 7).

Treatments for *M. abscessus* diseases are very challenging since *M. abscessus* is naturally resistant to β-lactams, tetracyclines, aminoglycosides, macrolides and to most anti-tubercular drugs (8, 9). Among the contributing factors to drug resistance, acquired drug resistance resulting from mutations in the drug targets has been reported (1, 10). In addition, the *M. abscessus* resistome encompasses various factors such as the low permeability of the cell envelope and the ability to modify antibiotics, converting them into unproductive metabolites (11). Expression of a large set of efflux pumps, including mycobacterial membrane protein large (MmpL) proteins, has been shown to participate in resistance to several drugs (12–14).

MmpL proteins are members of the resistance-nodulation-cell division superfamily of transmembrane transporters (15). Besides their participation in drug efflux, MmpL proteins can translocate complex, virulence-associated envelope lipids and siderophores across the plasma membrane to the periplasmic space using the proton motive force (PMF) (16, 17). MmpL protein members are present in high abundance in *M. abscessus* as compared to *M. tuberculosis* and a clear role of MmpLs in lipid transport and pathogenesis has been assigned to a few of these (16, 18–20). MmpL4a and MmpL4b, for instance, are responsible for the transport of surface-associated glycopeptidolipids (GPL) (21–24). These lipids are present in the smooth (S) morphotype, while they are absent in the rough (R) morphotype (22). They are essential for pathogenicity, motility, aggregation, biofilm formation, and contact with host cells (18, 22, 25–27). Moreover, the mycomembrane contains also several families of trehalose-containing lipids that can play a crucial role in these processes (28).

**Fig 1:**
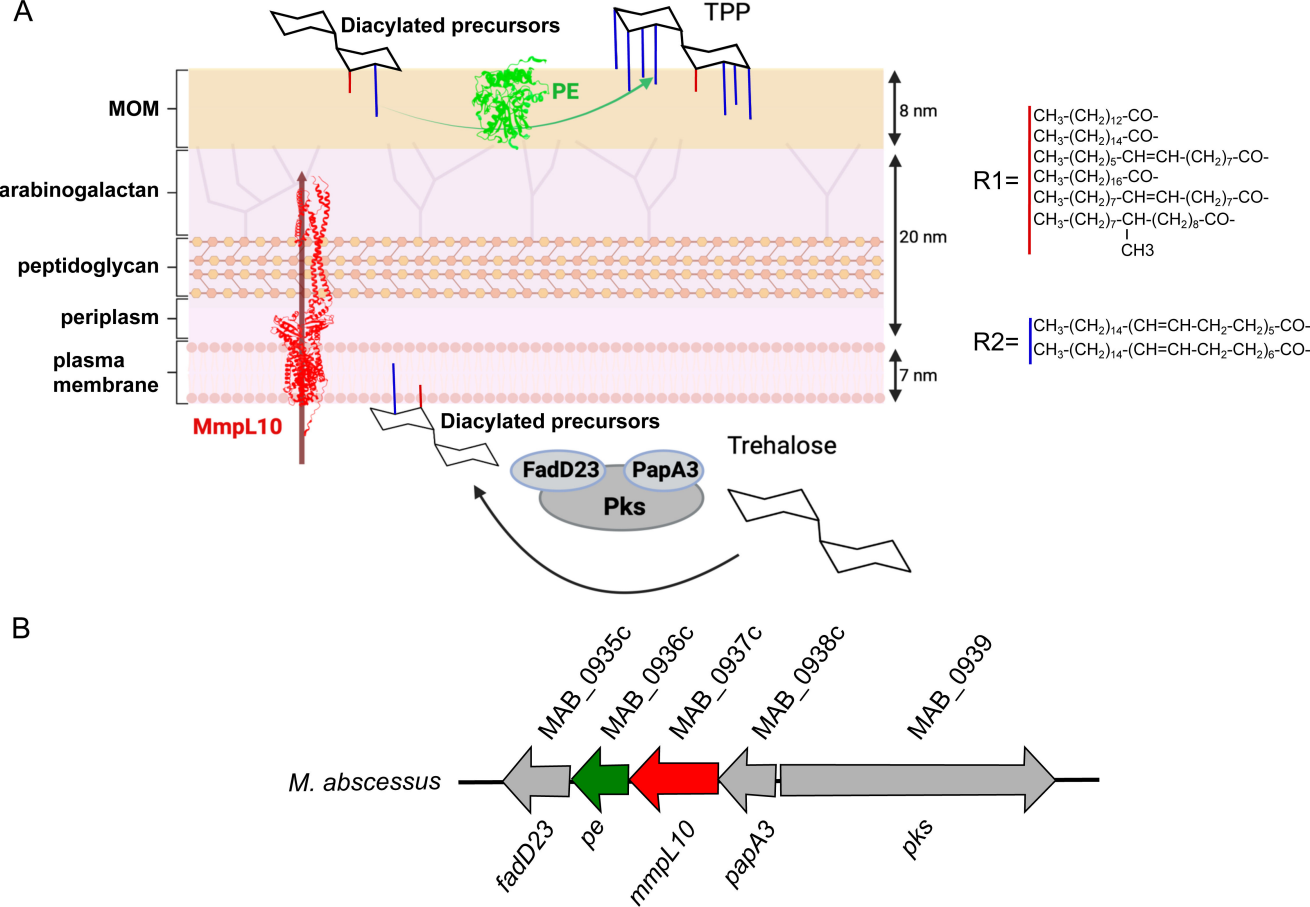
Representation of the TPP pathway and genomic organization. (**A**) TPPs consist of trehalose-bearing 6-7 polyunsaturated fatty acyl substituents (phleic acids) and one straight-chain fatty acid residue. TPP production starts in the cytosol with the formation of a 2,3-diacyl trehalose intermediate, containing a C14-C19 fatty acyl chain and a C36-C40 phleic acid substituent. The structures of R1 and R2 are derived from *M. smegmatis* (30). The diacylated trehalose precursors are then translocated across the plasma membrane *via* MmpL10 and exported to the cell surface, where the PE protein transacylates phleic acids (**R2**) between precursors to yield TPP. The structures of MmpL10 and PE were predicted using AlphaFold, and their dimensions were measured with UCSF Chimera. (**B**) Representation of the *M. abscessus* TPP locus.

Mycolic acids, the signature lipids of mycobacteria, are transported to the cell wall as trehalose esters by MmpL3 (19, 29). Additional cell wall-associated trehalose-based lipids, designated trehalose polyphleates (TPPs), have recently been reported in *M. abscessus* (30) (Fig. 1). MmpL10 is encoded by a *TPP* locus including five genes, which are required for the synthesis and transport of TPPs to the outer surface of the cell (30) (Fig. 1A and B). TPPs are high-molecular-weight, surface-exposed glycolipids and structurally related to sulfolipids SL-1 and polyacylated trehalose PAT found in *M. tuberculosis* (31). TPP production starts in the cytoplasm with the formation of diacyltrehalose precursors, which are translocated across the plasma membrane by MmpL10 and subsequently exported to the cell surface, where PE transacylates phleic acids between precursors to form TPP (30, 32). Although the periplasmic loop of MmpL10 is predicted to be nearly 10 nm in length, spanning most of the cell envelope, it remains unclear whether the diacyltrehalose intermediates are directly handed over from MmpL10 to PE or if additional proteins are involved in this process (Fig. 1A). Very little is known about the function of TPPs. Recently, it was shown that the clinically useful phages BPs and Muddy require TPPs for their infection of *M. abscessus* and *M. smegmatis*, putting into light the importance of TPPs in the efficacy of these two therapeutic phages (33). It was previously suggested that TPPs are required for the formation of *M. abscessus* cords (34), which represent an important virulence determinant characterizing the R variant of *M. abscessus* (35). Together, these observations point out a potential role for TPPs in the virulence of *M. abscessus,* while there is currently no report examining if the loss of TPPs on the surface of *M. abscessus* affects the outcome of the infection.

Here, we investigated the role of MmpL10 and TPPs in *M. abscessus* virulence by combining *in vitro*, *in cellulo,* and *in vivo* studies. Deletion of *mmpL10* resulted in reduced sliding motility and altered biofilm formation. Importantly, Δ*mmpL10* and Δ*pE* mutants showed the importance of TPPs in the survival of *M. abscessus* in macrophages and zebrafish embryos. Mechanistically, the absence of TPPs leads to a decreased ability to block phagosomal acidification after internalization by macrophages. Overall, this study provides the first insights into the biological function of TPPs and represents an important step toward our understanding of *M. abscessus* infection and virulence.

## RESULTS

### The TPP transporter activity of MmpL10 is dependent on its periplasmic coiled-coil motif and a functional PMF

**Fig 2:**
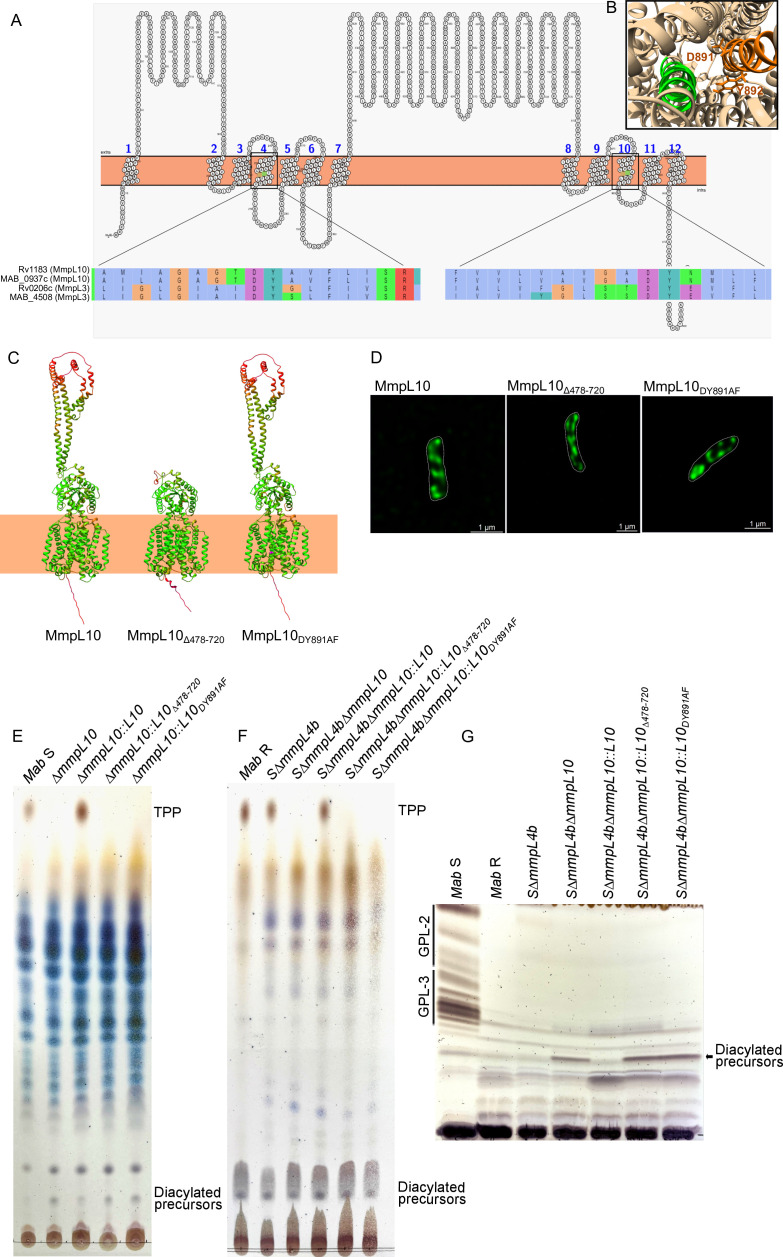
Generation of *mmpL10* mutants and complementation strains in *M. abscessus* (*Mabs*) S and R. (**A**) Topology prediction and multiple sequence alignments of the TM4 and TM10 regions of MmpL3 and MmpL10 from *M. abscessus* and *M. tuberculosis* highlight the strict conservation of Asp and Tyr residues (highlighted in purple and dark green in the sequence) in TM4 and TM10. (**B**) Bottom view of the AlphaFold prediction of MmpL10. TM4 is highlighted in green, and TM10 in orange. (**C**) AlphaFold prediction of the MmpL10 full-length protein, MmpL10_Δ478-720_ (lacking the extramembrane domain), and MmpL10_DY891AF_ (where the conserved Asp/Tyr pair has been replaced by Ala/Phe). The color code represents the accuracy of the prediction from green (very good prediction) to red (uncertain prediction). (**D**) Representative confocal microscopy images of Δ*mmpL10*-derived strains expressing either MmpL10-mNeonGreen, MmpL10Δ_478-720_-mNeonGreen, or MmpL10_DY891AF_-mNeonGreen to illustrate protein localization. A series of Z-stacks were collected with a Zeiss LSM880 Airyscan confocal microscope and the images were assembled and reconstructed with Zen blue software. (**E**) Thin-layer chromatography (TLC) analysis of total lipids extracted from an *mmpL10* deletion mutant of *M. abscessus* S, the parent S strain, and the mutant strain complemented with the full-length protein (**L10**), *mmpL10*_Δ*478-720*_ or *mmpL10_DY891AF_*. Eluent: CHCl_3_/CH_3_OH (90:10, vol/vol). TLC plates were revealed by spraying 0.2% anthrone on the plate, followed by charring. (**F**) TLC analysis of total lipids extracted from *M. abscessus* R, the parental strain *M. abscessus* S Δ*mmpL4b* (Δ*mmpL4b*; R morphotype) and its derivatives lacking *mmpL10* (Δ*mmpL10*) and complemented with either the full-length protein (Δ*mmpL10::L10*), *mmpL10*_Δ478-720_ or *mmpL10*_DY891AF_. (**G**) GPL profiles from the strains described in (**F**). *M. abcesssus* S was included as the GPL-producing control strain.

A conserved Asp/Tyr pair in the transmembrane domains TM4 and TM10 has been identified as essential for the functionality of several MmpL transporters, including MmpL3_Mtb_, by facilitating the MmpL proton pathway (18). Sequence alignment of TM4 and TM10 from MmpL3 and MmpL10 in *M. abscessus* and *M. tuberculosis*, respectively, reveals the presence of the conserved Asp/Tyr pair in MmpL10_Mab_ at positions 259 and 891 (Fig. 2A and B). Additionally, AlphaFold predictions of MmpL10_Mab_ highlight an extra-membrane coiled-coil-like motif, which may be involved in transporting TPP precursors across the plasma membrane (Fig. 2C). Deletion of this domain (MmpL10_Δ478-720_) or the introduction of point mutations in the TM10 Asp/Tyr pair (MmpL10_DY891AF_) did not alter the predicted overall protein structure (Fig. 2C). To verify whether these changes in MmpL10 affect its localization within the bacterial cell, we generated an *mmpL10* deletion mutant and subsequently complemented it with different *mmpL10* alleles fused to mNeonGreen. The *mmpL10* deletion mutant was first engineered in the S variant of *M. abscessus* CIP104536^T^ reference strain *via* homologous recombination (12), leading to S Δ*mmpL10* (Fig. S1A). PCR and sequencing analyses confirmed the predicted deletion of *mmpL10* (Fig. S1B). Fluorescence imaging showed that the localization of MmpL10, MmpL10_Δ478-720_, or MmpL10_DY891AF_ (Fig. 2D) was consistent with the previously reported subcellular distribution of MmpL10 (36).

Next, to verify the role of the MmpL10 transporter in the TPP pathway, S Δ*mmpL10* was complemented with full-length and mutated *mmpL10* alleles, each fused with a C-terminal HA-tag, using site-specific integration at the chromosomal *attB* site (37). The expression of each HA-tagged protein was validated by Western blotting (Fig. S1C).

While attempts to directly delete *mmpL10* in the rough (R) variant of CIP104536^T^ were unsuccessful, the R Δ*mmpL10* mutant was obtained by deleting the *mmpL4b* gene [*MAB_4115c,* encoding the MmpL4b GPL transporter (24)] in S Δ*mmpL10*, resulting in the double mutant Δ*mmpL4b/*Δ*mmpL10* with an R morphotype. As for S Δ*mmpL10*, complementation was validated by Western blotting (Fig. S1D). Consistent with mutations observed in clinical strains of *mmpL10* (33), total lipid extraction and thin-layer chromatography (TLC) analyses revealed the absence of TPPs in the *mmpL10* deletion mutants compared to the *M. abscessus* S, R, S Δ*mmpL4b* (R morphotype) progenitors, and the production of TPPs in the respective complemented strains carrying an intact copy of *mmpL10* (Fig. 2E and F). Moreover, the deletion of *mmpL10* in *M. abscessus* S led to the accumulation of the diacyltrehalose precursors (Fig. 1A and 2E), as reported (30). As anticipated, the deletion of *mmpL4b* resulted in the loss of GPLs, as confirmed by TLC analysis (Fig. 2G), as shown earlier (24). Complementing Δ*mmpL10* with an *mmpL10* allele harboring point mutations at D891 and Y892 within TM10 (Δ*mmpL10::mmpL10_DY891AF_*) resulted in the loss of TPPs and accumulation of the diacyltrehalose precursors (Fig. 2E), similar to Δ*mmpL10*, confirming the critical role of these residues in the MmpL10 transport function. A comparable phenotype was observed when the periplasmic loop from residues 478–720 was deleted (Δ*mmpL10::mmpL10_Δ478-720_*) (Fig. 2E and F).

Overall, these results imply that the loss of TPPs in Δ*mmpL10* mutants is caused by the absence of the MmpL10 transporter activity.

### Loss of TPP modestly alters *M. abscessus* growth, sliding motility, and biofilm formation

**Fig 3:**
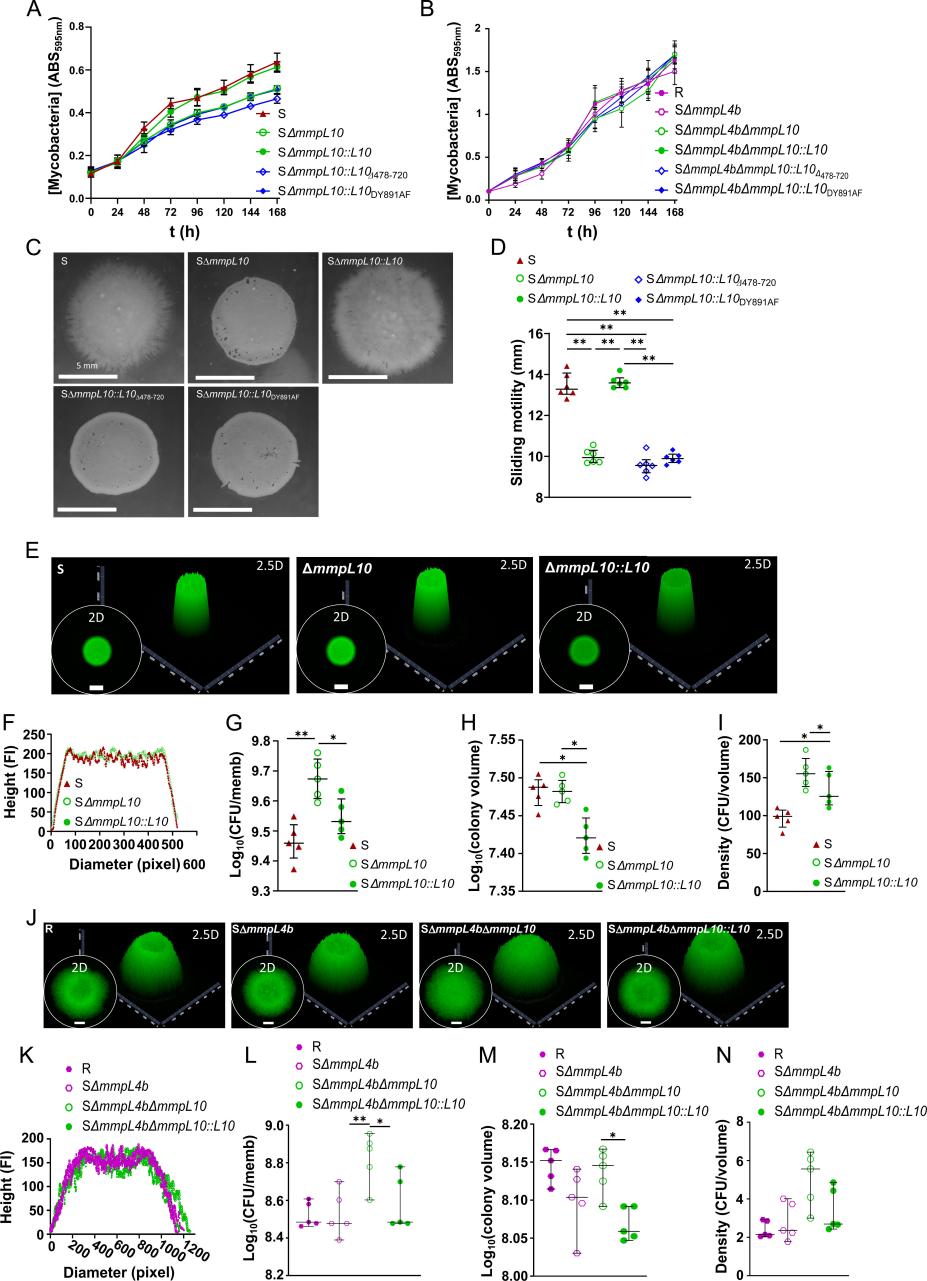
Effects of *mmpL10* deletion on bacterial growth, sliding, and biofilm formation. (**A and B**) *In vitro* growth of the *mmpL10* mutant and derivatives in the S (**A**) or R (**B**) genetic backgrounds. The experiment was performed using two biological replicates with six technical replicates per biological replicate. (**C**) Deletion of *mmpL10* in the S background modifies the sliding motility. The sliding motility experiment was performed using three biological replicates with two technical replicates per biological replicate. (**D**) Sliding motility diameter (in mm). (**E**) Representative 2- and 2.5-dimensional pictures of colony-biofilms of the S morphotype of *M. abscessus* on biofilm-supporting membranes after 5 days of incubation. The white scale bar indicates 5 mm. (**F–I**) Deletion of *mmpL10* alters the S morphotype colony-biofilm development (**F**). The CFU per membrane is shown in (**G**), the colony volume in (**H**), and the colony density in (**I**). (**J**) Representative 2- and 2.5-dimensional pictures of colony-biofilms of the R (Δ*mmpL4b*) background of *M. abscessus* on biofilm-supporting membranes after 5 days of incubation. The white scale bar indicates 5 mm. (**K–N**) Deletion of *mmpL10* alters the R morphotype colony-biofilm development (**K**). The CFU per membrane is shown in (**L**), the colony volume in (**M**), and the colony density in (**N**). The colony-biofilm experiment was performed using five biological replicates. The error bar denotes the interquartile range. *P < 0.05 and **P < 0.01.

Although TPPs are high-molecular-weight, hydrophobic, and surface-exposed glycolipids, their absence due to *mmpL10* mutations did not affect the hydrophobicity of either the S (Fig. S2A) or the R (Fig. S2B) strains. However, modest changes in growth, sliding motility, and biofilm formation were observed. All S strains lacking TPPs (S Δ*mmpL10*, S Δ*mmpL10::mmpL10*_Δ_*_478-720_*, and S Δ*mmpL10::mmpL10_DY891AF_*) showed a slightly altered planktonic growth pattern (Fig. 3A). However, the absence of *mmpL10* in R-derived strains resulted in no obvious change in growth compared to the S Δ*mmpL4b* strain (i.e., R morphotype) (Fig. 3B). Deletion of *mmpL10* reduced sliding motility in the S variant (Fig. 3C and D) and modestly impacted biofilm development in both S (Fig. 3E through I) and R (Fig. 3J through N) variants. S Δ*mmpL10* showed a 64% increase in the number of bacteria per membrane (Fig. 3G), leading to higher density (Fig. 3I) compared to the parental S strain. The S Δ*mmpL10::mmpL10* strain displayed a partial phenotype. In the R variant, the Δ*mmpL10* mutant exhibited a modest 1.5-fold increase in the number of bacteria per membrane (Fig. 3L) compared to the parental strain, with the R complemented strain behaving similarly to the R parental strain.

### TPPs play a critical role in intracellular survival

**Fig 4:**
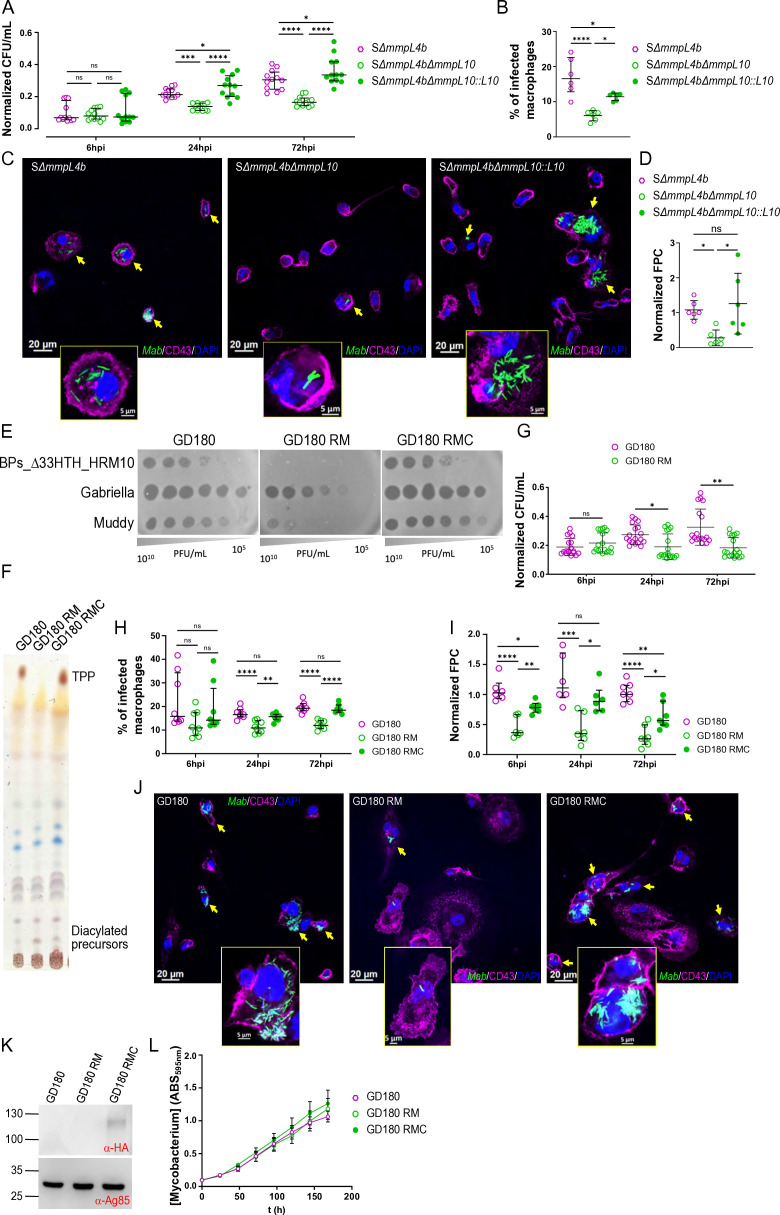
Reduced intracellular growth of TPP-deficient strains in macrophages. (**A**) Intracellular CFUs of the *mmpL10* deletion mutant of *M. abscessus* S Δ*mmpL4b* (R morphotype), the parental and the mutant strain complemented with the full-length protein (**L10**) were determined at 6, 24, and 72 hpi. CFUs were normalized to the respective inoculum. Data are median ± interquartile range for at least three independent experiments (each time in quadruplicate). Statistical analysis was performed using ordinary one-way analysis of variance (ANOVA) with Šídák’s multiple comparisons test. (**B**) Percentage of infected THP-1 macrophages at 72 hpi. Data are median ± interquartile range for three independent experiments. Statistical analysis was performed using ordinary one-way ANOVA with Šídák’s multiple comparisons test. (**C**) Three immunofluorescent fields were taken at 72 hpi using a confocal microscope (40× magnification), showing the macrophages infected with the various strains (in green). The nuclei are shown in blue, and the CD43 protein associated with the plasma membrane of macrophages is in magenta. Bacilli are inside the infected macrophages. (**D**) Bacterial load quantified by fluorescent pixel counts (FPC). Data were normalized to the S Δ*mmpL4b* and are shown as individual technical replicates within at least three independent experiments, with the median and interquartile range. Statistical analysis was performed using the Kruskal-Wallis test with Dunn’s multiple comparisons test. (**E**) Ten-fold serial dilutions of BPs_∆33HTH_HRM10, Gabriella, and Muddy were spotted onto solid media with *M. abscessus* clinical isolate GD180, the resistant mutant (GD180 RM), and the complemented strain (RMC). (**F**) TLC analysis of total lipids extracted from GD180, GD180 RM, and GD180 RMC. Eluent: CHCl_3_/CH_3_OH (90:10, vol/vol). TLC plates were revealed by spraying 0.2% anthrone on the plate, followed by charring. (**G**) Intracellular CFUs of GD180 and GD180 RM were determined at 6, 24, and 72 hpi. CFUs were normalized to the respective inoculum. (**H**) Percentage of infected THP-1 macrophages by the GD180-derived strains at 6, 24, and 72 hpi. Data are median ± interquartile range for three independent experiments. If the data were normal ordinary one-way ANOVA with Šídák’s multiple comparisons test was used, for non-normal data Kruskal-Wallis test with Dunn’s multiple comparisons test was used. (**I**) Bacterial loads were quantified by FPC. Data were normalized to the GD180 and are shown as individual technical replicates within at least three independent experiments, with the median and interquartile range. Statistical analysis was performed using the Kruskal-Wallis test with Dunn’s multiple comparisons test. (**J**) Three immunofluorescent fields were taken at 72 hpi using a confocal microscope (40× magnification), showing the macrophages infected with the various strains (in green). The nuclei are shown in blue, and the CD43 protein associated with the plasma membrane of macrophages is in magenta. Bacilli are inside the infected macrophages. (**K**) Overexpression of MmpL10-HA in GD180 RMC. Immunoblotting of full-length MmpL10 expression in the complemented strain using anti-HA antibodies (upper panel). The Ag85 protein complex was used as a loading control (lower panel). (**L**) Disruption of *mmpL10* in GD180 was associated with no growth change *in vitro*. The experiment was performed using two biological replicates with six technical replicates per biological replicate.

To analyze the contribution of MmpL10 to *M. abscessus* virulence, THP-1 macrophages were infected with Δ*mmpL10* and its respective wild type and complemented strains. Macrophages were lysed at 6-, 24-, and 72 hours post-infection (hpi) to determine the intracellular bacterial burden. For the R morphotype, the absence of TPPs resulted in a significant attenuation of bacterial survival compared to the wild-type strain (Fig. 4A). While the bacterial burden of the wild-type strain increased at 24 hpi and 72 hpi, the mutant replicated at a much slower rate. Functional complementation with an intact *mmpL10* gene (Δ*mmpL10::L10*) fully restored the wild-type phenotype (Fig. 4A). These results were confirmed by quantifying infected macrophages (Fig. 4B) using fluorescence microscopy at 72 hpi (Fig. 4C), revealing a marked reduction in the number of infected cells and their bacterial burden as determined by fluorescent pixel counts (Fig. 4D). To examine whether this effect is restricted to the reference strain, similar infection studies were conducted using the previously reported clinical strain GD180 isolated from a CF patient as well as GD180 RM, harboring a frameshift mutation in *mmpL10*. This mutation led to resistance against the therapeutic phages BPs and Muddy (33) (Fig. 4E) and the loss of TPPs (Fig. 4F), compared to its parental GD180 and complemented GD180 RMC counterparts. As for the CIP104536^T^ reference strain, a similar reduction in bacterial loads at both 24 and 72 hpi was observed after infection of THP-1 cells with GD180 RM (Fig. 4G through J). The reintroduction of a functional *mmpL10* gene into GD180 RM, confirmed by Western blotting (Fig. 4K), restored the intracellular bacterial burden to levels comparable to GD180 (Fig. 4H through J). Notably, the *in vitro* growth of all three strains was similar (Fig. 4L). This indicates that the intracellular growth defect is not specific to the reference strain but can also be observed in a clinical strain.

The same tendency was observed in macrophages infected with the S CIP104536^T^-derived mutant (Fig. S3). S Δ*mmpL10* exhibited a severe defect in intracellular survival in THP-1 macrophages compared to the S progenitor as early as 6 hpi (Fig. S3A). This decrease in bacterial burden was partially attributed to a defect in bacterial adhesion, as confirmed by the attachment assay (Fig. S3B), which showed that the mutant adhered slightly less than the parental strain. Quantification of infected macrophages after fluorescence microscopy also confirmed a significantly lower infection rate for S Δ*mmpL10* compared to the parental strain at 72 hpi, indicating reduced bacterial survival (Fig. S3C through E). The reduced bacterial burden persisted at 24 and 72 hpi (Fig. S3A). Complementation with an intact *mmpL10* gene (Δ*mmpL10::mmpL10*) fully restored the wild-type phenotype, while complementation with non-functional *mmpL10* alleles (Δ*mmpL10::mmpL10_Δ478-720_* or Δ*mmpL10::mmpL10_DY891AF_*) phenocopied the deletion mutant.

To distinguish whether the decreased intracellular viability of S Δ*mmpL10* is due to the loss of TPPs or the absence of the transporter itself, a deletion mutant of the acyltransferase PE was constructed (Fig. S1E and F). In Δ*pE,* the diacyltrehalose precursors are transported to the bacterial surface but cannot be transformed into TPPs (Fig. 1A; Fig. S4A) (32). Δ*pE* showed a similar impairment of bacterial survival as Δ*mmpL10* (Fig. S4B), with a limited growth defect during the Log phase *in vitro* (Fig. S4C). This supports the critical TPP requirement for *M. abscessus* intramacrophage survival. Although complementation with an intact copy of *pE* (Fig. S4D) resulted in the production of significantly lower amounts of TPPs compared to the wild-type strain, perhaps due to a partial polar effect of the *pE* disruption on the downstream expression of *fadD23* (Fig. S4A), this reduction was sufficient to fully restore the phenotype in macrophages. Like S Δ*mmpL10* in the attachment assay, S Δ*pE* was also defective in adhesion to macrophages compared to the parental strain (Fig. S4E).

Overall, these findings underscore the important role of TPPs in *M. abscessus* intracellular survival and infection process.

### TPP-defective mutants are associated with enhanced phagosome acidification and phagosome membrane damage in macrophages

**Fig 5:**
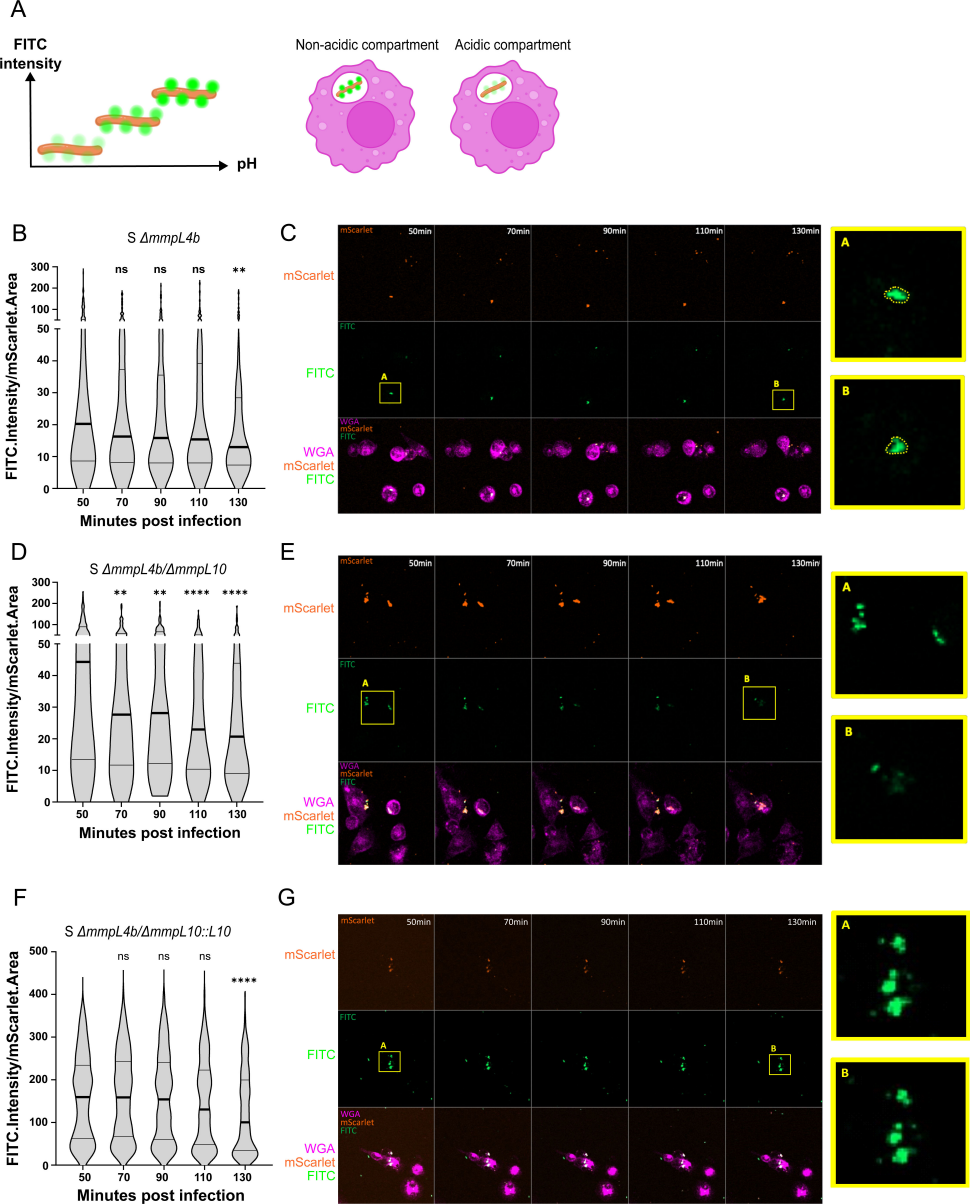
TPPs are involved in blocking phagosome acidification. (**A**) The FITC intensity is dependent on the pH. (**B–G**) Evolution of the FITC intensity during infection with S Δ*mmpL4b* (parental strain) (**B and C**), S Δ*mmpL4b/*Δ*mmpL10* (**D and E**), and complemented strain (S Δ*mmpL4b/*Δ*mmpL10::L10*) (**F and G**). The graphs (**B–D–F**) represent the ratio of the FITC.Intensity and the respective mScarlet.Area. Data are represented as a violin plot with the median and interquartile range. Each time point was compared to the first (50 min post-infection), using a two-tailed Mann-Whitney test. (**C–E–G**) Representative images of segmented events for each time point. Each column corresponds to a time point (from 50 min post-infection to 130 min post-infection) and each line is a channel. The yellow rectangle represents the zoomed-in images on the right, with the FITC channel.

Next, we investigated the mechanism by which TPPs may interact with macrophages. First, we asked whether a functional MmpL10 transporter and thus the presence of TPPs, inhibits phagosomal acidification, a crucial event for pathogenic mycobacteria to survive inside macrophages (38). Based on a previous report (25), we adapted an assay using mScarlet fluorescent bacilli labeled with the green fluorescent dye fluorescein (FITC) before infecting THP-1 macrophages (outlined in Fig. S5). FITC, which increases in fluorescence intensity with rising pH, allowed us to monitor intracellular pH levels (39) (Fig. 5A). Confocal imaging and segmentation tracked the FITC intensity of intracellular red fluorescent bacteria for 130 min, starting 50 min post-infection (Fig. 5B through G). No acidification was observed at early time points for the S Δ*mmpL4b* parental strain (Fig. 5B and C). In contrast, S Δ*mmpL4b/*Δ*mmpL10* (i.e., R morphotype) exhibited a decrease in FITC intensity over time (Fig. 5D and E), indicating that the bacteria are localized in a compartment that is progressively acidifying. This suggests that the reduced intracellular survival of S Δ*mmpL4b/*Δ*mmpL10* may be linked to phagosome acidification and subsequent bacterial degradation. Complementation of the mutant restored the phenotype to resemble that of the parental strain (Fig. 5F and G).

**Fig 6:**
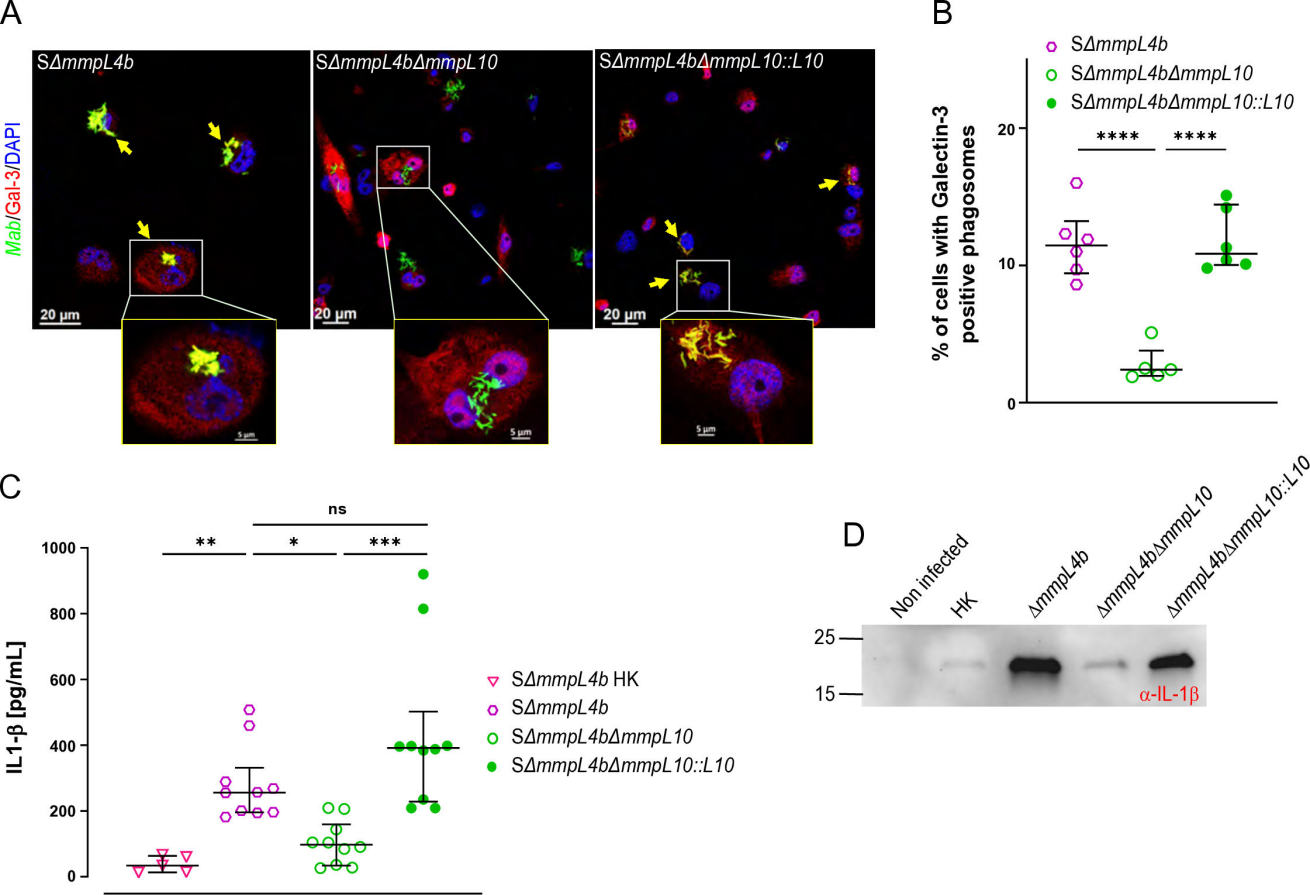
TPPs mediate communication between the phagosome and cytosol in *M. abscessus*. (**A**) MmpL10/TPP-dependent accumulation of Gal-3 based on the phagosomal lysis marker Gal-3 (red) occurs on phagosomes containing *M. abscessus* (green) after 20 hpi. *M. abscessus* is visualized by its autofluorescence upon excitation at a green wavelength. Quantification of the results is shown in (**B**). (**C**) IL-1β production during THP-1 infection. The response to infection with an *mmpL10* deletion mutant of *M. abscessus* S Δ*mmpL4b*, the parental and complemented strain was assessed by quantifying IL-1β secretion. Histograms with error bars represent the median ± interquartile range for at least three independent experiments. HK stands for heat-killed. (**D**) IL-1β secretion associated with the *mmpL10* deletion mutant of *M. abscessus* S Δ*mmpL4b*, the parent and complemented strain was analyzed by Western blot using IL-1β antibodies. Statistical analysis was performed using the Kruskal-Wallis test with Dunn’s multiple comparisons test. ns, non-significant; *P < 0.05; **P < 0.01; ***P < 0.001; ****P < 0.0001.

Surviving mycobacteria have the ability to escape the phagosome, resulting in cytosolic communication and activation of cytosolic pathways (40). To evaluate TPP’s role in phagosomal membrane damage, we analyzed phagosomal membrane disruption using Galectin-3 (Gal-3) staining (41). At 20 hpi, colocalization of S Δ*mmpL4b/*Δ*mmpL10* with Gal-3 was significantly lower than that of the S Δ*mmpL4b* or the complemented strain, indicating a decreased ability to damage the phagosomal membrane and to reach the cytosol for the TPP-defective strain (Fig. 6A and B). To confirm Δ*mmpL10’s* inability to establish cytosolic connections, we assessed IL-1β production, a biomarker of cytosolic *M. tuberculosis* and *M. abscessus* (42, 43). While S Δ*mmpL4b* infection induced high levels of secreted IL-1β from macrophages, the mutant resulted in significantly less IL-1β secretion, as determined by enzyme-linked immunosorbent assay (Fig. 6C) and Western blotting (Fig. 6D). Similar observations were made with the S morphotype (Fig. S6).

Overall, this indicates that the inactivation of MmpL10 with loss of TPPs reduces *M. abscessus’* ability to block phagosome acidification, which may accelerate bacterial degradation rather than triggering phagosomal escape and establishing cytosolic connections and induction of the IL-1β pathway.

### TPP-defective *M. abscessus* shows slight bacterial growth impairment and reduced pathogenicity in zebrafish

**Fig 7:**
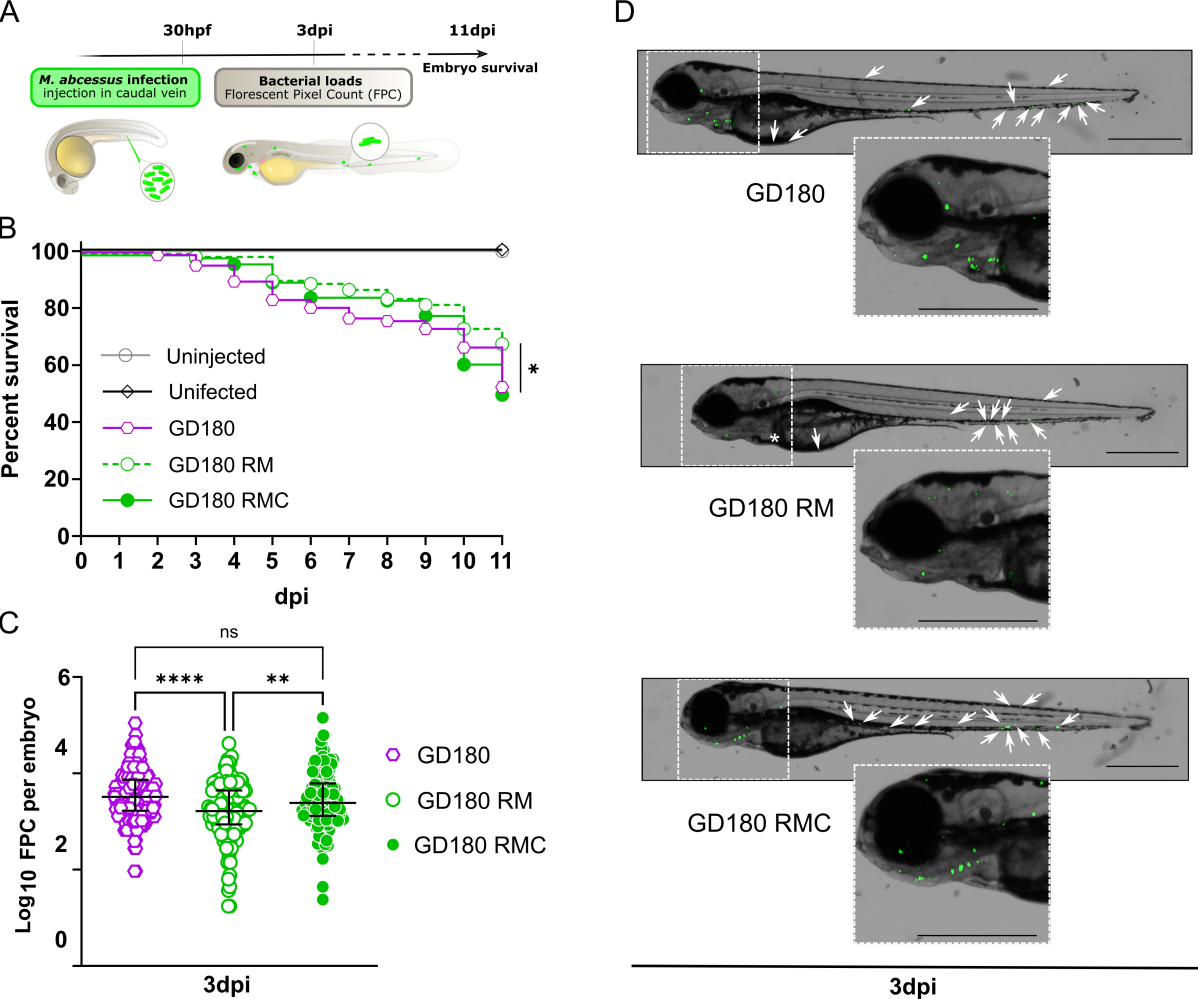
TPP-deficient *M. abscessus* shows reduced virulence in zebrafish embryos. (**A**) Schematic representation of the protocol showing the timeline of zebrafish embryo infected *via* caudal vein injection (700–900 CFU) of *M. abscessus* GD180, GD180 RM, and GD180 RMC expressing mWasabi. (**B**) Survival curves GD180, GD180 RM, and GD180 RMC infected zebrafish embryos were monitored over 11 dpi. Survival curves are the cumulative results of four experiments covering more than 24 infected larvae per group. Statistical analysis was performed using the log-rank (Mante-Cox) statistical test (*P < 0.05). (**C**) Bacterial loads of infected embryos at 3 dpi. The bacterial burden was quantified by FPC determination using the ImageJ software. Results are expressed in log_10_ FPC, with each data point representing a single embryo. Values for all data displayed are the mean ± SD with the merge of three independent experiments. Statistical analysis for FPC quantification was performed using the Kruskal-Wallis multiple comparison test with Dunn’s correction (**P < 0.01; ****P < 0.0001; ns, not significant). (**D**) Whole embryo imaging at 3 dpi following infection with GD180, GD180 RM, and GD180 RMC. Scale bars represent 600 µm. Enlargement of the head in the left panel (zoom in 1.8×). *, indicates autofluorescence. White arrows indicate small infection foci.

The zebrafish embryo is an excellent model for studying the pathophysiology of *M. abscessus* (18, 27, 35, 44). Given that only R variants induce acute and lethal infections in this model, we injected 30 h post-fertilization embryos in the caudal vein with the above-mentioned R clinical *M. abscessus* strains GD180, the GD180 RM and its complemented strain GD180 RMC. Embryo survival was monitored daily for 11 days, and bacterial burden was assessed at 3 days post-infection (dpi) (Fig. 7A). While infection with GD180 gradually led to larval death, reaching 50% mortality by 11 dpi, GD180 RM showed slight attenuation in virulence, with 30% killing by 11 dpi (Fig. 7B). Reintroduction of the wild-type *mmpL10* allele into GD180 RM restored virulence to levels comparable to GD180 (Fig. 7B). The reduced embryo mortality was corroborated by a decreased bacterial burden for GD180 RM compared to GD180 at 3 dpi, as assessed by fluorescent pixel count (FPC), indicating reduced bacterial replication and attenuation of GD180 RM in zebrafish (Fig. 7C and D). GD180 RMC partially restored the wild-type phenotype (bacterial loads and embryo killing). Contrary to earlier reported observations suggesting that TPPs are involved in cord formation (34), our findings demonstrate that cord formation occurs within zebrafish embryos regardless of the presence or absence of TPPs (Fig. S7A). Furthermore, cords were also visualized *in vitro* based on colony morphology, where the GD180 RM strain was capable of forming rough and corded colonies in different media (Fig. S7B). In addition, both extracellular and intracellular cord formation by GD180 RM were observed following infection of THP-1 macrophages (Fig. S7C). This indicates that the ability of *M. abscessus* to form cords is maintained even in the absence of TPPs both in the extracellular environment and within the infected host.

Overall, these results in zebrafish align with observations in macrophages and support the view that TPPs contribute to *M. abscessus* virulence in the infected host.

## DISCUSSION

Although TPPs are not present in *M. tuberculosis,* the proteins encoded by the *TPP* locus display sequence similarities with enzymes required for the production of sulfolipids (SL), diacyltrehaloses (DAT), and polyacyltrehaloses (PAT), which are closely related in structure to TPPs and known to participate in the pathogenicity of *M. tuberculosis* (28, 45). While the biosynthetic pathway of TPPs has been discovered recently, their biological function in mycobacterial physio(patho)logy remains unknown. We show here that *M. abscessus* requires TPPs for survival in macrophages and virulence in a zebrafish model of infection. By generating genetically defined *mmpL10* as well as *pE* mutants, we demonstrate that TPP loss results in (i) a reduced sliding motility, (ii) an enhanced biofilm formation *in vitro,* and (iii) impaired intramacrophage survival.

*M. abscessus* produces a broad range of surface-exposed lipids with major biological functions and an exceptionally large repertoire of MmpL transporters (16), whose respective substrates and role in virulence were first reported in *M. tuberculosis* (46). In *M. abscessus,* the importance of MmpL4 in GPL transport and the S-to-R transition upon *mmpL4* deletion has been well described (18, 22, 23). Moreover, MmpL8_MAB_ is required for the proper expression of a new glycolipid family and is important for the intracellular survival of *M. abscessus* within macrophages and zebrafish (20) whereas MmpL3, the essential trehalose monomycolate transporter, represents an attractive drug target for future drug developments (19, 47). As *M. abscessus* has a very large number of MmpL proteins, it is believed that some of them have additional functions, such as detoxification or drug efflux mechanisms (12, 13). However, MmpL10 does not seem to act as an efflux pump as no differences in drug susceptibility were observed between *mmpL10* mutants and their respective progenitors (Table S1).

**Fig 8:**
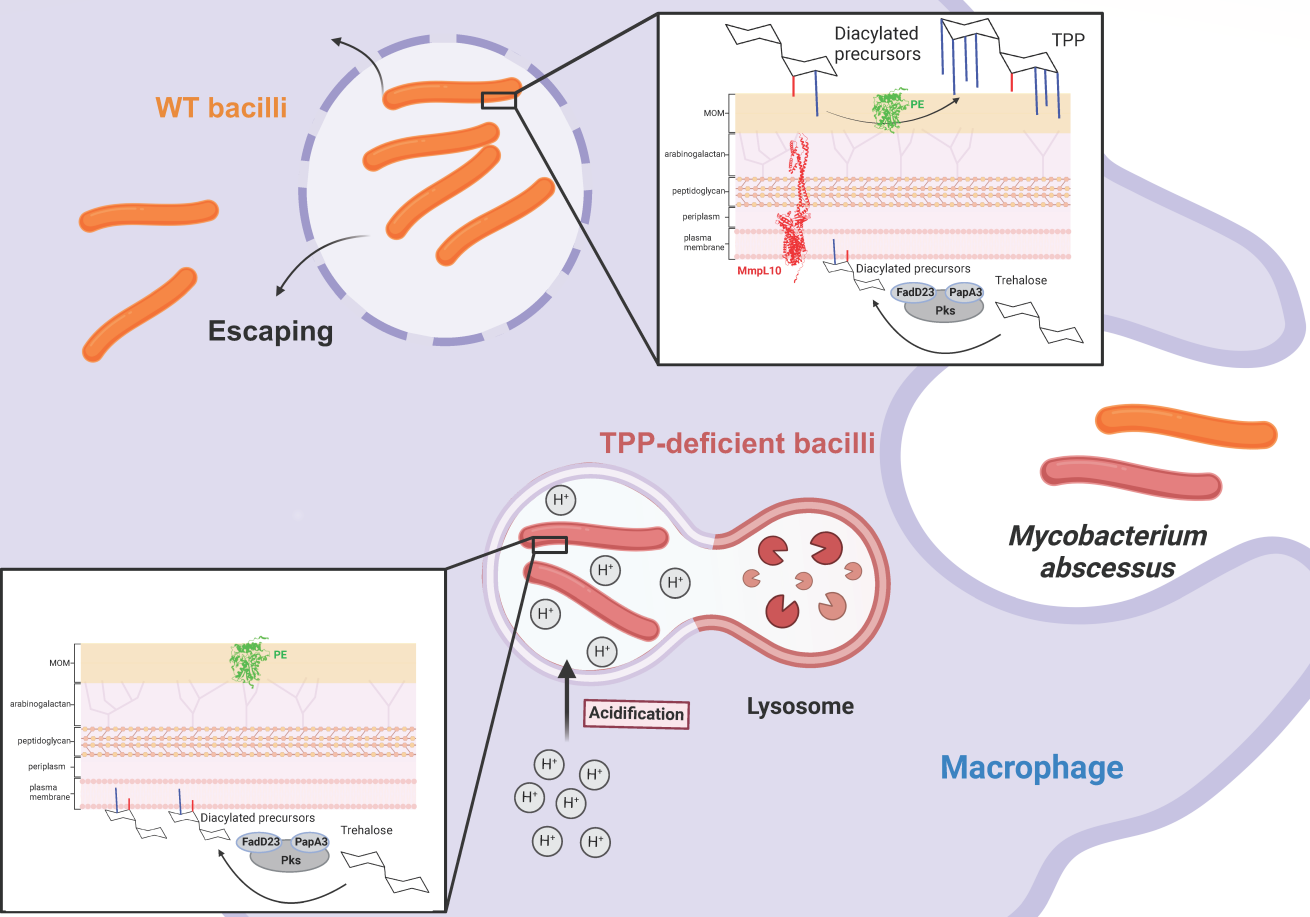
Effects of TPP depletion on *M. abscessus* pathogenesis. *M. abscessus* produces diacyltrehalose precursors in the cytoplasmic compartment. After translocation across the inner membrane by MmpL10 and presumably by additional partners that remain to be identified, these intermediates are further acylated by PE to generate TPPs. Following infection, WT bacilli are internalized in macrophage phagosomes, block fusion with acidic compartments, escape the phagosome, enter the cytosol where they trigger the inflammasome, multiply and disseminate. In contrast, TPP-deficient mutants (lacking either *mmpL10* or *pE*) fail to block the phagolysosomal fusion process. In these mature phagosomes, the mutants cannot resist the acidic pH and are more rapidly eliminated by the macrophage.

As shown for *M. smegmatis* (30)*,* we confirmed that deletion of *mmpL10* in S and R variants of *M. abscessus* abrogated TPP production, which was rescued upon gene complementation. Moreover, we show that infection of human THP-1 cells with the Δ*mmpL10* mutants was associated with decreased intramacrophage survival and a reduced proportion of infected cells. After internalization by macrophages, it has been shown that the R variant of *M. abscessus* is able to escape into the cytosol *via* phagosomal rupture to avoid degradation after phagolysosomal fusion (40). Here, we observed that R Δ*mmpL10* has an impaired capacity to block phagosomal acidification and was unable to co-localize with Galectin-3, a marker of phagosomal membrane damage. This suggests that TPPs participate, directly or indirectly, in phagolysosomal fusion and in phagosomal membrane damage to establish cytosolic communication (Fig. 8). The cytosolic access of the R variant through phagosomal damage leads to enhanced cytokine production, such as IL-1β and type I interferon and cell death, which resulted in their cell-to-cell spreading (40). This seems to be impaired when TPPs are absent. However, the possibility cannot be excluded that the attenuation of TPP-deficient mutants is also linked with a decreased capacity for macrophage internalization, contributing to the reduced intracellular bacterial burden. Further investigations are required to determine whether the TPP mutants are less efficiently taken up by these cells.

We could also confirm the importance of TPPs by demonstrating that the lack of TPPs affects the fitness and virulence of a clinical *M. abscessus* strain in zebrafish embryos. However, we noted that disruption of *mmpL10* in GD180 only partially attenuated the strain as evidenced by the modest reduction in embryo killing and bacterial burden. Future experiments will be conducted to determine the virulence status of this mutant in mouse models of infection. Contrary to previous observations suggesting that TPPs are involved in cord formation (34), based on the fact that extraction of the cell surface-associated lipid fraction (containing TPPs) by petroleum ether resulted in the loss of corded bacilli, we could observe cord formation *in vitro*, in infected macrophages as well as in zebrafish embryos, regardless of the presence or absence of TPPs.

In addition to the attenuation in intracellular survival, we found that deletion of *mmpL10* slightly impaired the sliding motility of *M. abscessus*. This may affect the invasion of *M. abscessus* lacking TPPs as the sliding motility is involved in abiotic and biotic surface colonization by mycobacteria both in the environment and in the host (48). Many studies had reported that this motility is mainly due to the presence of GPL on the surface of *M. abscessus* (22, 27, 49). Here, we note that the deletion of *mmpL10* favored a modest increase in the number of mycobacteria and cellular density inside *M. abscessus* colony-biofilms. Biofilm formation is a hallmark of various *M. abscessus* infections, e.g., device-related infections or lung infections in patients with underlying pathologies (50). Like any other bacterium, *M. abscessus* develops biofilms under unfavorable conditions (51), and these biofilms exhibit greater intrinsic resistance to antimycobacterial drugs (52) and phagocytosis (53) than their planktonic form. As previously noted, Δ*mmpL10* mutants grown under planktonic conditions show reduced intramacrophage survival, suggesting that increased biofilm formation may serve as a compensatory mechanism to resist phagocytosis. Similar adaptive mechanisms have been described in antibiotic-susceptible *Escherichia coli* strains isolated from prosthetic joint infections (54) and enterococci from urinary tract infections (55), both of which showed greater biofilm-forming capacity than their antibiotic-resistant counterparts. The reasons for the higher bacterial density within Δ*mmpL10* biofilms remain unknown and warrant further studies.

Although TPPs are dispensable for *in vitro* growth, the TPP pathway does not represent a valuable therapeutic target to restrict *M. abscessus* growth *in vitro*. However, since the loss of TPPs compromises the intracellular fitness of *M. abscessus*, one could speculate that chemical inhibition of TPP synthesis or transport would reduce both bacterial burden and pathology *in vivo*. Thus, MmpL10 and other proteins involved in the TPP synthesis and transport pathway represent potential targets for new therapeutic developments. In this respect, we showed that, like in MmpL4 or MmpL3 (18), the conserved Asp/Tyr pair in TM10 is a key element in the MmpL proton pathway and is essential for the functionality of MmpL10 in *M. abscessus*. Thus, MmpL10 could be targeted in a similar way as described for MmpL3 (18, 56) by blocking the PMF. Another target might be the coiled-coil motif of MmpL10 located in the periplasm and absent in MmpL3. However, further studies are needed to investigate how this extension is involved in TPP transport toward the cell wall and whether it represents a platform of interaction with additional proteins required for the translocation of the diacyltrehalose precursors to the outer membrane.

TPPs are required for infection of BPs and its derivatives as well as Muddy, with important implications for clinical use, as both of these have been used therapeutically (57). A simple explanation for the role of TPPs is that they act as receptors for recognition by BPs and Muddy and the other TPP-dependent phages, with phenotypes that are consistent with a TPP requirement for adsorption to the bacterial surface (33). In the first case of the therapeutic use of mycobacteriophages, BPs, and Muddy were used in combination with ZoeJ, and it is of interest that ZoeJ does not display TPP-dependence. Unexpectedly, resistance to BP derivatives or Muddy has not been observed in clinical use, even in 11 cases where Muddy was used alone (57, 58). Thus, it appears highly likely that resistance through TPP loss *in vivo* is not observed due to the trade-off of reduced fitness and poor virulence of TPP-defective strains, as shown in this study.

In summary, our study highlights the importance of TPPs as new surface lipids contributing to the pathogenicity of *M. abscessus*. It sheds new light on the role of complex lipids in the interaction between mycobacteria and host cells. The wide distribution of TPPs across mycobacterial species suggests that they may also exhibit important pathogenic functions in other clinically important opportunistic species, such as *M. avium*. Chemical inhibition of TPP synthesis/transport may offer new therapeutic opportunities, particularly for the treatment of cystic fibrosis patients with NTM diseases. Overall, this work helps to address the significant knowledge gap regarding the basic biology of NTM cell envelope lipids (59).

## MATERIALS AND METHODS

### Bacterial strains, plasmids, and primers

Strains, plasmids, and primers are listed in Tables S2 to S4, respectively.

### Culture conditions

Rough (R) and smooth (S) variants of *M. abscessus* CIP104536^T^ (60) and related mutants were grown at 37°C in Middlebrook 7H9 broth (BD Difco) supplemented with 0.025% Tyloxapol (Sigma-Aldrich) and 10% oleic acid, albumin, dextrose, catalase (OADC enrichment; BD Difco) (7H9^T/OADC^), on Middlebrook 7H10 agar supplemented with OADC (7H10^OADC^) or on LB agar (Roth) plates containing the appropriate antibiotics. When required, kanamycin (250 µg/mL, Sigma-Aldrich) or hygromycin (1 mg/mL, Sigma-Aldrich) were added to the media.

### Deletion of *mmpL10* and *pE* genes

Unmarked deletion of the endogenous *mmpL10* or *pE* gene was performed in the merodiploid strain using a strategy developed previously (12). Briefly, the pUX1-*katG-mmpL10/pE* was designed to generate an unmarked deletion of the corresponding open reading frames. After the two steps of selection of the homologous recombination events, the DNA junctions were PCR amplified and sequenced to confirm the proper genotype of the mutants using primers listed in Table S4. While this strategy failed in the rough morphotype, the R Δ*mmpL10* mutant was obtained by deleting the *mmpL4b* gene (*MAB_4115*c) in S Δ*mmpL10* using the pUX1-*katG-mmpL4b*. The unmarked deletion of *mmpL4b* was confirmed by PCR amplification and sequencing. Thus, the R Δ*mmpL10* genotype corresponds to S Δ*mmpL10*/*mmpL4b*.

### Complementation and overexpression of MmpL10 or PE

Complementation plasmids were generated using the integrative pMV306 harboring the *tet* operator sequence (tetO-4C5G) (61). Genes of interest fused to an HA-tag were PCR-amplified using the Q5 High-Fidelity DNA Polymerase (New England Biolabs) and *M. abscessus* genomic DNA. Primers were designed to introduce an HA tag at the 3′ ends of the genes of interest. Linear fragments were purified on agarose gels (NucleoSpin Gel and PCR Clean-up, Macherey-Nagel). Following the manufacturer’s instructions, In-Fusion SNAP Assembly Master Mix (Takara) reactions were done to insert the linear fragments into the integrative pMV306 (insertion at the *attL5* mycobacteriophage insertion site in the *glyV* tRNA gene). The resulting plasmids were introduced into Stellar competent cells (Takara), verified by sequencing, and electroporated into *M. abscessus*. To monitor the bacteria *in vivo*, green versions of the resulting plasmids were generated by adding the *mWasabi* coding sequence under the control of the constitutive Pleft* promoter, and red versions by adding the *mScarlet* coding sequence. The Pleft* element is a derivative of the P*left* promoter from mycobacteriophage L5 (62) associated with a ribosomal binding site that increases expression levels (63).

### CFU determination of infected macrophages

To assess intracellular CFU at 6, 24, and 72 hpi, macrophages were washed once with 1× PBS and lysed with 100 µL 1% Triton X100. Lysis was stopped by adding 900 mL 1× PBS and serial dilutions were plated to monitor the intracellular bacterial counts. CFU were counted after 3 days of incubation at 37°C.

### Immunofluorescence staining of infected macrophages

For microscopy-based infectivity assays, THP-1 cells were cultured on coverslips in 24-well plates at a density of 10^5^ cells/well and incubated for 72 h at 37°C with 5% CO_2_. Cells were infected with mWasabi-expressing strains (MOI 2:1) for 4 h, washed, treated with amikacin, and fixed at 72 hpi with 4% paraformaldehyde in PBS for 15 min. Cells were permeabilized with 0.2% Triton X-100 for 20 min. After blocking with 2% BSA in 1× PBS supplemented with 0.2% Triton X-100 for 15 min, cells were incubated with an anti-CD43 antibody (Becton Dickinson; dilution 1:1,000) for 1 h, followed by an Alexa Fluor 594-conjugated anti-mouse secondary antibody (Invitrogen). Subsequently, cells were stained with 1 µg/mL 40,6-diamidino-2-phenylindole (DAPI; Becton Dickinson) for 5 min, washed with 1× PBS, and mounted onto microscope slides using Immumount (Calbiochem). Images were acquired by capturing combined signals of CD43 (magenta) and *M. abscessus* (green) using a Zeiss Axioimager confocal microscope equipped with a 40× or 63× oil objective, with image processing performed using Zeiss Axiovision software. Quantification of intracellular bacilli was done using ImageJ, with consistent imaging and scoring parameters applied across all samples. The percentage of infected macrophages was determined starting at 6 hpi, a time point at which only bacilli that have successfully entered macrophages can be detected. Bacteria that either failed to attach, were not properly internalized, or were killed early by macrophages are not included in the quantification. Confocal microscopy enabled the precise detection of bacilli within macrophages, allowing for the differentiation between infected and uninfected cells. The percentage of infected macrophages was calculated by counting macrophages containing internalized bacilli (green) compared to the total number of macrophages, which were identified by CD43 staining (magenta). High-resolution confocal microscopy images (extracellular cords, infected, and uninfected macrophages) were acquired using a Zeiss LSM880 confocal microscope with an Airyscan module and a plan-Apochromat 63× Oil 1.4 NA DIC M27 objective. For visualization of the MmpL10 protein, mNeonGreen bacilli were sectioned (Z-stacks) and slices were acquired every 0.18 µm, resulting in 12 serial slices per image. 3D imaging reconstruction of the Airyscan confocal microscopy images was performed with ZEN Blue 3.7.

### Bacterial load determination of infected macrophages

For determination of bacterial load using FPC, cells were seeded in PhenoPlate 96-well microplates with black walls and cyclic olefin bottoms (2 × 10^4^ cells/well) and labeled with Wheat-Germ-Agglutinin (WGA), Alexa Fluor 647 conjugate after differentiation. Infection was performed as described above. Epifluorescence images of 80% of the wells were acquired using a Cell-Discoverer 7 microscope (Carl Zeiss SAS, France) with a Plan-Apochromat 5×/0.35 objective and 1× tubulens optovar, using Tiles (12 scenes/well). Images were segmented using Zen Blue software (Zeiss) as follows: cells were identified as a class of objects using WGA segmentation and intracellular bacteria were identified as a subclass within the cells using the mWasabi segmentation. For each segmented subclass the product of mWasabi.Area and mWasabi.intensitySum was normalized to the respective inoculum, which was determined by measuring the total mWasabi signal right after infection. The percentage of infected macrophages was determined using the same segmentation with the count parameter.

### Attachment assay

THP-1 monocytes were grown and differentiated as described above. Before infection, THP-1 macrophages were placed on ice for 30 min. Infection with *M. abscessus* (MOI 100:1) was performed for 30 min on ice to prevent phagocytosis. Plates were centrifuged for 1 min at 1,200 rpm to facilitate sedimentation of bacteria onto the cells. To remove non-adherent bacteria, infected THP-1 cells were washed 3 times with cold 1× PBS. Subsequently, 900 µL of cold, sterile water was added and cells were incubated for 40 min on ice. Cells were lysed by adding 100 µL 1% Triton X100. Serial dilutions were plated to monitor the intracellular bacterial counts. CFU were counted after 3 days of incubation at 37°C.

### Acidification assay

Wild type (S Δ*mmpL4b*), Δ*mmpL10,* and complemented strains expressing pMV306-mScarlet were labeled with 100 µg/mL FITC (Sigma-Aldrich). THP-1 cells were seeded in PhenoPlate 96-well microplates with black walls and cyclic olefin bottoms (2 × 10^4^ cells/well). After differentiation, cells were labeled with WGA, Alexa Fluor 647 conjugate and infected with the different FITC-labelled *M. abscessus* strains at an MOI of 100 in PBS supplemented with CaCl_2_ (0.9 mM) + MgSO_4_ (0.5 mM) + 1% FBS for 30 min at 37°C and 5% CO_2_. To remove extracellular bacteria, cells were washed 3× with PBS, and the plate was placed in a Cell-Discoverer 7 microscope (Carl Zeiss SAS, France). Confocal images were acquired with a Plan-Apochromat 20×/0.7 objective and 0.5× tubulens optovar, every 20 min for 6 cycles, using Tiles (72 scenes/well) to cover around 1,000 cells. Images were segmented using the Zen Blue software (Zeiss) as follows: cells were identified as a class of objects using WGA segmentation, intracellular bacteria were identified as a subclass within the cells using the mScarlet segmentation, and finally, the FITC labeling was segmented as a sub-subclass within the bacterial population. For each segmented bacterium, the FITC.Intensity was divided by the mScarlet.Area and plotted for every time point.

### Galectin-3 immunostaining

THP-1 cells were seeded on coverslips in 24-well plates at a density of 10^5^ cells/well. Cells were infected with mWasabi-expressing *M. abscessus* (MOI 10:1) for 4 h, washed, treated with amikacin, and fixed at 20 hpi with 4% paraformaldehyde in PBS for 15 min. Phagosomal membrane damage was detected by immunostaining using a purified mouse anti-human Galectin-3-specific Mab (555746; BD Pharmingen) at a 1/1,000 dilution and a 594-conjugated anti-mouse secondary antibody (Invitrogen). Images were acquired by focusing on combined signals (Gal-3 in red and *M. abscessus* in green) and captured on a Zeiss Axioimager confocal microscope equipped with a 40× or 63× oil objective and processed using Zeiss Axiovision software. An infected cell was considered positive if at least one mycobacteria-containing phagosome was stained positive for the phagosome damage marker. Most specific Gal-3-positive signals were associated with membranous structures surrounding bacteria-containing phagosomes. The percentages of infected cells with at least one *M. abscessus*-containing phagosome positive for Gal-3 were determined from at least 900 infected cells.

### Statistical analysis

Statistical analysis was performed on Prism 10.2.2 (Graphpad, La Jolla, CA, USA) and detailed in each figure legend. The normality of data was evaluated using a Shapiro-Wilk test. Descriptive data are cited as median and interquartile range in case of non-normal distribution. A non-parametric Kruskal-Wallis test was used to compare more than three groups and a Wilcoxon test was used to compare two groups. Zebrafish survival assays are represented in Kaplan-Meier graphs and analyzed with a Log-rank test. The significance between multiple selected groups was determined using a one-way analysis of variance (ANOVA) with Šidák’s Multiple Comparisons test after validating the normality of the data. Significance is indicated as ns, non-significant; *P < 0.05; **P < 0.01; ***P < 0.001; ****P < 0.0001.

## Data Availability

All data generated in this study are available upon request.

## SUPPLEMENTAL MATERIAL

Supplemental Material mbio.02970-24-s0001.pdf Supplemental text, tables, and figures.

ASM does not own the copyrights to Supplemental Material that may be linked to, or accessed through, an article. The authors have granted ASM a non-exclusive, world-wide license to publish the Supplemental Material files. Please contact the corresponding author directly for reuse.
