# Supplementary material for: Trehalose polyphleates participate in Mycobacterium abscessus fitness and pathogenesis
Source: mBio. 2024 Oct 30;15(12):e02970-24. doi: 10.1128/mbio.02970-24 (PMC11633156; doi:10.1128/mbio.02970-24)
Supplement: Supplemental Material — Supplemental text, tables, and figures. [file mbio.02970-24-s0001.pdf]

SUPPORTING INFORMATION

Trehalose polyphleates participate in *Mycobacterium abscessus* fitness and pathogenesis

Silke Malmsheimer[1], Wassim Daher[1,2,$], Yara Tasrini[1,$], Claire Hamela[1,$], John Jairo Aguilera-Correa[1], Christian Chalut[3], Graham F. Hatfull[4,*] and Laurent Kremer[1,2,*]

[1]Centre National de la Recherche Scientifique UMR 9004, Institut de Recherche en Infectiologie de Montpellier (IRIM), Université de Montpellier, 1919 route de Mende, 34293, Montpellier, France.
[2]INSERM, IRIM, Montpellier, France.
[3]Institut de Pharmacologie et de Biologie Structurale (IPBS), Université de Toulouse, CNRS, Université Toulouse III – Paul Sabatier (UT3), Toulouse, France
[4]Department of Biological Sciences, University of Pittsburgh, Pittsburgh, PA 15260, USA

[$]Equal contribution

*Corresponding author: Graham Hatfull: gfh@pitt.edu
Laurent Kremer: laurent.kremer@irim.cnrs.fr

Running title: TPPs are virulence lipids in *M. abscessus*

Keywords: *Mycobacterium abscessus*, trehalose polyphleates, MmpL10, infection, pathogenesis, macrophage, zebrafish.

Supplementary Materials and Methods

Growth curves

Pre-cultures in exponential phase were diluted to reach an optical density (OD) at 595nm (OD_{595nm}) of 0.05 in 7H9 broth supplemented with 10% OADC, 0.2% glycerol, and 0.025% Tyloxapol and dispensed in 96-well microtiter plates (200 µL/well). Plates were incubated at 37 °C without agitation and measurements were taken daily using a Tecan Spark 10M multimode microplate reader (Tecan).

Sliding motility

Sliding motility of each strain was evaluated using a modified methodology previously described (1). Ten µL of 24 hrs cultures (OD_{600nm} = 0.9–1.0) were dropped in the centre of well from a 6-well containing 7H9 broth with 0.3% agar and without adding any carbon source. The sliding distance was measured after for 10 days at 37 °C under humidified conditions.

Colony-biofilm model

Mycobacterial biofilms were produced based on a recently developed method (2). *M. abscessus* cultures were grown in $7H9^{OADC}$ containing 0.2% glycerol, and tyloxapol (0.025%) at 37 °C and 80 rpm for 72 hrs. Cultures were centrifuged at 5,000 rpm for 5 min, washed two times with sterile PBS, and then diluted to an OD of 0.5 (~1.5×10^8 CFU/mL). Autoclaved black, polycarbonate membranes (diameter, 25 mm; pore size, 0.2 µm; Whatman, Merck, Darmstadt, Germany) were placed on $7H11^{OADC}$ and inoculated with 10 µL of the bacterial suspension. The membrane-supported biofilms were statically incubated for 5 days at 37 °C and humidity. Membrane-supported biofilms were imaged and processed using a binocular (ZEISS Axio Zoom.V16, Zeiss) equipped with a lighting device (Zeiss HXP 200C) at 7× zoom, detecting mWasabi fluorescence. On biofilm fluorescence pictures, a straight line was drawn (covering all the diameter of the biofilm), and intensity fluorescence of each pixel was retrieved to draw fluorescence spectra using the ZEN 2 software (Blue Edition). Colony-biofilms were removed from the plates with sterile clamps, deposited in a 50 mL tube containing 10 mL PBS supplemented with 0.025% tyloxapol and a mixture of 5 mm- and 1 mm-diameter glass beads, vortexed for 15 s, and sonicated until complete disaggregation of pieces of colony was macroscopically considered. The resulting bacterial suspension was diluted in a 10-fold bank dilution in PBS supplemented with tyloxapol and plated on $7H11^{OADC}$ agar. Plates were incubated at 37 °C for 4 days prior to CFU counting.

Hydrophobicity assay

Mycobacterial surface hydrophobicity was determined following a modification of the methodologies described previously (3). Each strain was seeded on $7H9^{OADC}$ at 37 °C for 72 hrs and colonies were resuspended and sonicated in 5 mL of 0.9% NaCl in PYREX® 16 × 100 mm disposable round-bottom threaded culture tubes (Corning) before measuring the OD_{595nm} (A_0) of 200 µL in triplicate was measured using spectrophotometer multimode microplate reader (Tecan Spark 10M). Subsequently, 2 mL of hexadecane (Sigma-Aldrich) was added and the content vortexed for 30 s. After a 20 min phase separation, the OD_{595nm} of 200 µL in triplicate was measured using the microplate reader. Subsequently the lower (aqueous) phase (A_1) was measured. The percentage of hydrophobicity (H) of each strain embedded in the organic hexadecane phase was estimated using the equation $H (\%) = [(A_0 – A_1)/A_0] \times 100$.

Antibiotic susceptibility testing

Antibiotic susceptibility was tested using broth microdilution following the European Committee on Antimicrobial Susceptibility Testing (EUCAST) (4). 96-well RAPMYCOI Sensititre™ titration plates (Thermo Fisher Scientific, Massachusetts, USA) were used following the manufacturer's recommendations. Experiment were done using two biological replicates.

Western blotting

Mycobacterial cultures were grown in 7H9 broth. Bacteria were harvested, washed, resuspended in cold phosphate buffer saline (PBS) containing a protease inhibitor cocktail (Sigma-Aldrich) and lysed by the addition of 1-mm-diameter glass beads followed by 3 min cycle pulses (twice) at full speed in a bead-beater device (Mixer Mill MM 301, Retsch). Lysates were collected and protein concentrations assessed using the BCA Protein Assay Reagent kit (ThermoFisher Scientific). Equal amounts of proteins were separated by 10% SDS-PAGE and transferred onto a polyvinylidene fluoride membrane (Immobilon-P, Merck Millipore). Membranes were probed with rat anti-HA Abs (1:5,000 dilution) or mouse monoclonal 32/15 Abs, recognizing the Ag85 complex (1:10 dilution) (5), washed and incubated with anti-rat or anti-mouse antibodies conjugated to horseradish peroxidase (1:5,000). Signals were detected using HRP reaction (SuperSignal West Femto, ThermoFisher Scientific) on a ChemiDoc MP system (Bio-Rad).

Total lipid extraction and TLC analysis

Bacteria were grown in LB medium at 37 °C without agitation and pelleted by centrifugation (3,000 *g*, 10 min, room temperature). Lipids were extracted from bacterial pellets treated successively with $CHCl_3/CH_3OH$ (1:2, v/v) and $CHCl_3/CH_3OH$ (2:1, v/v), washed with water and dried. Lipids were resuspended in $CHCl_3$ and spotted on silica gel G60 plates (10×20 cm, Macherey-Nagel). Lipids were separated with $CHCl_3/CH_3OH$ (90:10, v/v) and detected by praying the plates with a 0.2% anthrone (Sigma-Aldrich) solution (w/v) in concentrated H_2SO_4 and charring.

GPL extraction

GPL were extracted from bacteria grown on 7H10^{OADC} agar plates without detergent, first with $CHCl_3/CH_3OH$/0.3%NaCl (9:10:3, v/v/v) and then by $CHCl_3/CH_3OH$/0.3%NaCl (5:10:4, v/v/v). The combined solvent extracts were mixed for 5 min with $CHCl_3$ and 0.3%NaCl (1:1, v/v) and centrifuged at 2,000 *x g* for 5 min to separate the lower-organic phase from the aqueous phase. The upper aqueous layer was discarded, and the lower-organic phase was evaporated under a stream of nitrogen and resuspended in chloroform/methanol (2:1, v/v). Polar lipids were subjected to TLC analysis and GPL separated using $CHCl_3/CH_3OH$/water (90:10:1, v/v/v). Orcinol/sulfuric acid vapor was used to visualize GPLs.

Preparation of *M. abscessus* inocula for infection studies

Due to the high propensity of *M. abscessus* R to form large bacterial aggregates and cords, specific treatments were applied to generate homogeneous and quantitatively controlled inocula prior to microinjections into embryos or infecting macrophages. This treatment applies also to smooth bacteria that produce smaller bacterial clusters. Briefly, exponentially growing bacteria were collected by centrifugation and resuspended in 1 mL PBS. Suspensions were homogenized with a 26-G needle (15 up-and-down sequences) and sonicated up for 1 min in a water bath sonicator. Fifty mL 7H9$^{T/OADC}$ were added to the suspended bacteria, filtered through 5 µm filters and bacteria were harvested after centrifugation and resuspended in 200 mL 7H9$^{T/OADC}$. Before proceeding with infections, the final bacterial concentration was assessed by plating serial dilutions onto LB agar and CFU counting after 3 days of incubation at 37 °C. Frozen inocula stocks (10 µL aliquots) were stored at -80 °C. For each experiment, CFU were assessed from aliquots frozen at -80 °C, a prerequisite to determine the exact number of viable bacteria to allow for reproducible use of bacteria from one experiment to another.

Cell culture and macrophage infection

The human pro-monocytic cell line THP-1 was maintained in RPMI 1640 glutamax supplemented with 10% fetal bovine serum (RPMI^{FBS}) and 1% penicillin/streptomycin (Gibco™) and were kept at 37 °C under an atmosphere containing 5% CO_2. Cells were differentiated into macrophages by adding 20 ng/mL Phorbol Myristate Acetate for 72 hrs now of seeding. Infection was performed in RPMI 1640 glutamax supplemented with 10% FBS for 4 hrs at 37 °C and 5% CO_2. The inoculum was plated on LB to assess bacterial viability. To kill extracellular bacteria, cells were washed once and treated for 2 hrs with 250 µg/mL amikacin in RPMI^{FBS}, washed 3 times and incubated in RPMI^{FBS} supplemented with 50 µg/mL amikacin.

IL-1β ELISA

Supernatants from THP-1/*M. abscessus* co-cultures (MOI 10:1) were collected at 20 hpi and assayed for human IL-1β secretion using the IL-1β kit (Biolegend), according to the manufacturer's instructions.

AlphaFold modeling

The structure of MmpL10 (MAB_0937c) was predicted using AlphaFold, based on its amino acid sequence. The PDB file of the highest-confidence model was exported. Chimera software was used to measure the protein dimensions and to visualize specific áreas through color-coding.

Zebrafish care and ethics statements

All zebrafish experiments were approved by the Direction Sanitaire et Vétérinaire de l'Hérault for the ZEFIX-CRBM zebrafish facility (Montpellier) (registration number C-34-172-39). Handling and experiments were approved by "le ministère de l'enseignement supérieur, de la recherche et de l'innovation" under the reference APAFIS#24406-2020022815234677 V3. Experiments were done using the *golden* mutant (6) crossed with wild-type AB zebrafish, maintained, as described earlier (7). Eggs were obtained by natural spawning, quickly bleached and incubated at 28.5 °C in Petri dishes containing E3 medium (5 mM NaCl, 0.17 mM KCl, 0.33 mM $CaCl_2$, 0.33 mM $MgSO_4$).

Zebrafish infection experiments

Experiments were done with *M. abscessus* GD180 (8), a mutant in *mmpL10* (GD180 RM) and its complemented derivative (GD180 RMC), all expressing mWasabi. At 24 hours post-fertilization (hpf), embryos were enzymatically dechorionated using 1mg/mL of pronase (stock of 5 mg/mL diluted in E3). At 30 hpf, embryos were anesthetized with 200 µg/mL of tricaine followed by caudal vein

microinjection of 3 nL of bacterial suspension (~250 CFU/nL for survival and FPC experiments or ~450 CFU/nL for cording observation at 3dpi) diluted in a strerile solution of polyvinylpyrrolidone (PVP, MW 10 000, Calbiochem) 2% in PBS supplemented with 0.05% phenol red for microinjection visualization. The size of the inoculum was verified *a posteriori* by injecting 3 nL of each bacterial suspension in sterile PBS and plated on $7H10^{OADC}$. To monitor embryo survival, infected larvae were transferred into 6-well plates (12 embryos/well) and incubated at 28.5 °C. Survival curves were determined by counting dead larvae at a daily basis for up to 11 days. Dead embryos were determined based on the absence of a heartbeat and removed from the well.

Zebrafish microscopy

For zebrafish imaging, embryos were anesthetized with 200 µg/mL of tricaine and immobilized with 3% methyl cellulose in a lateral position. Images were acquired with an EVOS™ M7000 Imaging System at 3dpi with the Olympus™ Objective PlanApo 2X, 0.08 NA or PL FL 10X LWD PH, 0.25NA/9.2WD using the EVOS™ Light Cube, GFP 2.0. After imaging, embryos were rinsed and transferred back the 6-well plate with fresh E3. Image processing was done using ImageJ.

Table S1. Susceptibility to drugs of *M. abscessus* S, Δ*mmpL10*, Δ*pE* and complementing strains using Sensititre RAPMYCO2 Susceptibility Testing Plates.

Antibiotic	Range (µg/mL)	MIC (µg/mL) (Phenotype) S	Δ*mmpL10*	Δ*mmpL10*::C
Trimethoprim/Sulfamethoxazole	0.25/4.75-8/152	>8/152(R)	>8/152(R)	>8/152(R)
Linezolid	132	32 (R)	16 (I)	16 (I)
Ciprofloxacin	0.12/4	>4 (R)	>4 (R)	>4 (R)
Imipenem	264	64 (R)	32(R)	64 (R)
Moxifloxacin	0.25-8	>8 (R)	>8 (R)	>8 (R)
Cefepime	132	>32 (R)	>32 (R)	>32 (R)
Cefoxitin	4-128	64 (I)	32 (I)	64 (I)
Amoxicillin/Clavulonic acid 2:1 ratio	2/1-64/32	>64/32 (R)	>64/32 (R)	>64/32 (R)
Amikacin	164	32 (I)	32 (I)	32 (I)
Ceftriaxone	464	>64 (R)	>64 (R)	>64 (R)
Doxycycline	0.12-16	>16 (R)	>16 (R)	>16 (R)
Minocycline	18	>8 (R)	>8 (R)	>8 (R)
Tigecycline	0.015-4	1 (S)	0.5 (S)	1 (S)
Tobramycin	116	16 (R)	16 (R)	16 (R)
Clarithromycin	0.06-16	4 (I)	4 (I)	4 (I)
Clarithromycin (at day 15)	0.06-16	>16 (R)	>16 (R)	>16 (R)

Antibiotic	Range (µg/mL)	MIC (µg/mL) (Phenotype) S	Δ*pE*	Δ*pE*::C
Trimethoprim/Sulfamethoxazole	0.25/4.75-8/152	>8/152(R)	>8/152(R)	>8/152(R)
Linezolid	132	32 (R)	32 (R)	32 (R)
Ciprofloxacin	0.12/4	>4 (R)	>4 (R)	>4 (R)
Imipenem	264	64 (R)	64 (R)	64 (R)
Moxifloxacin	0.25-8	>8 (R)	>8 (R)	>8 (R)
Cefepime	132	64 (R)	64 (R)	64 (R)
Cefoxitin	4-128	>64 (I)	>64 (I)	>64 (I)
Amoxicillin/Clavulonic acid 2:1 ratio	2/1-64/32	>64/32 (R)	>64/32 (R)	>64/32 (R)
Amikacin	164	32 (I)	16 (S)	32 (I)
Ceftriaxone	464	>64 (R)	>64 (R)	>64 (R)
Doxycycline	0.12-16	>16 (R)	>16 (R)	>16 (R)
Minocycline	18	>8 (R)	>8 (R)	>8 (R)
Tigecycline	0.015-4	1 (S)	0.5 (S)	1 (S)
Tobramycin	116	16 (R)	16 (R)	>16 (R)
Clarithromycin	0.06-16	2 (S)	2 (S)	2 (S)
Clarithromycin (at day 15)	0.06-16	>16 (R)	>16 (R)	>16 (R)

[a]The phenotype was attributed according to the breakpoints for rapidly-growing mycobacteria (9).
S: susceptible. R: resistant. I: intermediate.

Table S2. Strains used in this study.

Strains and Plasmids	Description	Source
Escherichia coli, strain Stellar™	*E. coli* HST08 strain that provides high transformation efficiency	Takara bio Cat. #636763
M. abscessus CIP104536^T Smooth strain (S)	Identifier: ATCC19977^T	Laboratoire de Référence des Mycobactéries (IP, France)
M. abscessus CIP104536^T Rough strain (R)	Identifier: ATCC19977^T	Laboratoire de Référence des Mycobactéries (IP, France)
CIP S::Pleft*, mWasabi	Kan^R derivative of CIP S in which mWasabi is constitutively expressed	This work
CIP S Δ*mmpL10*::Pleft*, mWasabi	Derivative of CIP S in which the native *mmpL10* is deleted. Expresses constitutively mWasabi	This work
CIP S Δ*mmpL10*::Pleft*, mWasabi-TetOFF, *mmpL10*	Hyg^R derivative of CIP S Δ*mmpL10* containing a copy of *mmpL10* (HA-tagged) integrated into the L5 attachment site. Expresses constitutively mWasabi	This work
CIP S Δ*mmpL10*::Pleft*, mWasabi-TetOFF,*mmpL10*$_{\Delta 478\text{-}720}$	Hyg^R derivative of CIP S Δ*mmpL10* containing a copy of *mmpL10*Δ478-720 (HA-tagged) integrated into the L5 attachment site. Expresses constitutively mWasabi	This work
CIP S Δ*mmpL10*::Pleft*, mWasabi-TetOFF,*mmpL10* $_{DY891AF}$	Hyg^R derivative of CIP S Δ*mmpL10* containing a copy of *mmpL10*DY891AF (HA-tagged) integrated into the L5 attachment site. Expresses constitutively mWasabi	This work
CIP S Δ*mmpL10*::TetOFF, *mmpL10*-mNeonGreen	Hyg^R derivative of CIP S Δ*mmpL10* containing a copy of the fusion protein *mmpL10-mNeonGreen* integrated into the L5 attachment site.	This work
CIP S Δ*mmpL10*::TetOFF, *mmpL10*$_{\Delta 478\text{-}720}$-mNeonGreen	Hyg^R derivative of CIP S Δ*mmpL10* containing a copy of the fusion protein *mmpL10*$_{\Delta 478\text{-}720}$-*mNeonGreen* integrated into the L5 attachment site.	This work
CIP S Δ*mmpL10*::TetOFF, *mmpL10*$_{DY891AF}$-mNeonGreen	Hyg^R derivative of CIP S Δ*mmpL10* containing a copy of the fusion protein *mmpL10*$_{DY891AF}$-*mNeonGreen* integrated into the L5 attachment site.	This work
CIP S Δ*pE*	CIP S in which the native *pE* is deleted.	This work
CIP S Δ*pE*::TetOFF, pE	Hyg^R derivative of CIP S Δ*pE* containing a copy of *pE* (HA-tagged) integrated into the L5 attachment site.	This work
CIP R::Pleft*, mWasabi	Kan^R derivative of CIP R, expresses mWasabi	This work
CIP S Δ*mmpL4b::*Pleft*, mWasabi	Kan^R derivative of CIP S in which the native *mmpL4b* is deleted, expresses mWasabi	This work

CIP S Δ*mmpL4b*/Δ*mmpL10*::Pleft*, mWasabi	Kan^R derivative of CIP S in which the native *mmpL4b* and *mmpL10* are deleted, expresses mWasabi	This work
CIP S Δ*mmpL4b*/Δ*mmpL10*::Pleft*, mWasabi,-TetOFF,mmpL10	Hyg^R derivative of CIP S Δ*mmpL4b*/Δ*mmpL10* containing a copy of *mmpL10* (HA-tagged) integrated into the L5 attachment site. Expresses mWasabi	This work
CIP S Δ*mmpL4b*::Pleft*, mScarlett	Kan^R derivative of CIP S in which the native *mmpL4b* is deleted, expresses mScarlett	This work
CIP S Δ*mmpL4b*/Δ*mmpL10*::Pleft, mScarlett	Kan^R derivative of CIP S in which the native *mmpL4b* and *mmpL10* are deleted, expresses mScarlett	This work
CIP S Δ*mmpL4b*/Δ*mmpL10*::Pleft*, mScarlett,-TetOFF,*mmpL10*	Hyg^R derivative of CIP S Δ*mmpL4b*/Δ*mmpL10* containing a copy of *mmpL10* (HA-tagged) integrated into the L5 attachment site. Expresses mScarlett	This work
GD180	*M. abscessus subsp. abscessus*, R variant	(8)
GD180 RM	*M. abscessus* subsp. *abscessus*, BPs resistant mutant, frameshift mutation in *mmpL10*, R variant.	(8)
GD180:Pleft, mWasabi	HygR derivative of GD180, expresses mWasabi	This work
GD180 RMC	Hyg^R derivative of GD180RM, expresses mWasabi	This work
GD180 RM:TetOFF, *mmpL10*	Hyg^R derivative of GD180RM, containing a copy of *mmpL10* (HA-tagged) integrated into the L5 attachment site. Expresses mWasabi	This work

Table S3. Plasmids used in this study.

Plasmid	Parent vector/ resistance	Cloning technique	Source of DNA, primers, restriction enzymes	Reference
pMV306-K-P_{left*} *mWasab*i	pMV361-K-L5 *attB*::P_{left*} *mWasabi* Kan^R	-	Source of P_{left*} *mWasab*i	(10)
pMV306-K-P_{left*} *mScarlett*	Kan^R	-	Source of P_{left*} *mScalett*	(10)
pMV306-H-Tet_{OFF} *mmpL3-HA*	pMV306-K-Tet_{OFF} *mmpL3-HA* Hyg^R	-	Source of pMV306-H backbone	(11)
pMV306-H-Tet_{OFF} *mmpL10-HA*	pMV306-H-Tet_{OFF} *mmpL3-HA* Hyg^R	In-Fusion cloning	PCR using pMV306-H-Tet_{OFF}-*mmpL3*-HA plasmid and MAB genomic DNA as templates with, respectively, pMV306-*hsp60* HA (Right)/ Inf P766 (R2) and 4c5GP_*mmpL10*_f/ HA_*mmpL10*_r primers and circularization by In-Fusion reaction	This work
pMV306-H-Tet_{OFF} *mmpL10*$_{\Delta 478\text{-}720}$-*HA*	pMV306-H-Tet_{OFF} *mmpL10-HA* *Hyg^R*	In-Fusion cloning	PCR using pMV306-H-Tet_{OFF} *mmpL10-HA* plasmid as templates with mmpL10-720down_f/ L10-720down_L10478up_r primers and circularization by In-Fusion reaction	This work
pMV306-H-Tet_{OFF} *mmpL10*$_{DY891AF}$-*HA*	pMV306-H-Tet_{OFF} *mmpL10-HA* *Hyg^R*	Site-directed mutagene sis	Site-directed mutagenesis of pMV306-H-Tet_{OFF} *mmpL10-HA* using QC_MAB0937c_D891AY892F_f QC_MAB0937c_Y892FD891A_r	This work
pMV306-H-P_{left*} *mWasabi*-Tet_{OFF} *mmpL10-HA*	pMV306-H-Tet_{OFF} *mmpL10-HA* Hyg^R	Restriction site cloning	Linearization of pMV306-K-P_{left*} *mWasab*i and pMV306-H-Tet_{OFF} *mmpL10-HA* by MfeI and SpeI plasmids and circularization by by T4 DNA Ligase	This work
pMV306-H-P_{left*} *mScarlett*-Tet_{OFF} *mmpL10-HA*	pMV306-H-Tet_{OFF} *mmpL10-HA* Hyg^R	Restriction site cloning	Linearization of pMV306-K-P_{left*} *mScarlett* and pMV306-H-P_{left*} *mWasabi*-Tet_{OFF} *mmpL10-HA* by NheI and XbaI plasmids and circularization by T4 DNA Ligase	This work
pMV306-H-P_{left*} *mWasabi*-Tet_{OFF} *mmpL10*$_{\Delta 478\text{-}720}$-*HA*	pMV306-H-Tet_{OFF} *mmpL10*$_{\Delta 478\text{-}720}$-*HA* Hyg^R	Restriction site cloning	Linearization of pMV306-K-P_{left*} *mWasab*i and pMV306-H-Tet_{OFF} *mmpL10*Δ478-720-*HA* by MfeI and SpeI plasmids and circularization by by T4 DNA Ligase	This work
pMV306-H-P_{left*} *mWasabi*-Tet_{OFF} *mmpL10*$_{DY891AF}$-*HA*	pMV306-H-Tet_{OFF} *mmpL10*$_{DY891AF}$-*HA* Hyg^R	Restriction site cloning	Linearization of pMV306-K-P_{left*} *mWasab*i and pMV306-H-Tet_{OFF} *mmpL10 DY891AF-HA* by MfeI and SpeI plasmids and circularization by by T4 DNA Ligase	This work
pMV306-H-Tet_{OFF} *mmpL10-mNeonGreen*	pMV306-H-Tet_{OFF} *mmpL10-HA* *Hyg^R*	In-Fusion cloning	PCR using pMV306-H-Tet_{OFF} *mmpL10-HA* and pMV306-eccC3-mNeonGreen plasmids with, respectively, linker_r/pmv306_f	This work

			and Linker_Neongreen_f/pmv306_Neongreen_r primers and circularization by In-Fusion reaction	
pMV306-H-Tet$_{OFF}$ *mmpL10*$_{\Delta 478\text{-}720}$-*mNeonGreen*	pMV306-H-Tet$_{OFF}$ *mmpL10*$_{\Delta 478\text{-}720}$-*HA* *Hyg^R*	In-Fusion cloning	PCR using pMV306-H-Tet$_{OFF}$ *mmpL10*Δ478-720-*HA* and pMV306-eccC3-mNeonGreen plasmids with, respectively, linker_r/pmv306_f and Linker_Neongreen_f/pmv306_Neongreen_r primers and circularization by In-Fusion reaction	This work
pMV306-H-Tet$_{OFF}$ *mmpL10*$_{DY891AF}$-*mNeonGreen*	pMV306-H-Tet$_{OFF}$ *mmpL10 DY891AF -HA* *Hyg^R*	Site-directed mutagenesis	PCR using pMV306-H-Tet$_{OFF}$ *mmpL10 DY891AF -HA* and pMV306-eccC3-mNeonGreen plasmids with, respectively, linker_r/pmv306_f and Linker_Neongreen_f/pmv306_Neongreen_r primers and circularization by In-Fusion reaction	This work
pMV306-H-Tet$_{OFF}$ *pE-HA*	pMV306-H Hyg^R	In-Fusion cloning	PCR using pMV306-H-Tet$_{OFF}$ *mmpL10-HA* plasmid and MAB genomic DNA as templates with, respectively, inf P766 (R2)/pmv306-hsp60 HA (Right) and tetoff_Mab0936c_f/HA_MAB0936c_rprimers and circularization by In-Fusion reaction	This work
pUX1-*katG-MAB_4106c*	Kan^R			(12)
pUX1-*katG-MAB_4115c* (*mmpL4b*)	pUX1-*katG-MAB_4106c* Kan^R			(11)
pUX1-*katG-MAB_0937c* (*mmpL10*)	pUX1-*katG-MAB_4106c* Kan^R	Restriction site cloning	Linearization of pUX1-*katG-MAB_4106c* by PacI-NheI; PCR using genomic DNA as template with MAB-0937c U PacI (F/ MAB-0937c U MfeI (R)and MAB-0937c D MfeI (F)/ MAB-0937c D NheI (R)primers, digested with PacI/MfeI or MfeI/NheI and cloned into linear pUX1-*katG* using T4 DNA ligase.	This work
pUX1-*katG-MAB_0936c* (*pE*)	pUX1-*katG-MAB_4106c* Kan^R	Restriction site cloning	Linearization of pUX1-*katG*-MAB_4106c by PacI-NheI; PCR using genomic DNA as template with MAB-0936c U PacI (F/ MAB-0936c U MfeI (R)and MAB-0936c D MfeI (F)/ MAB-0936c D NheI (R)primers, digested with PacI/MfeI or MfeI/NheI and cloned into linear pUX1-*katG* using T4 DNA ligase.	This work

Table S4. Primers used in this study.

Name	Sequence 5' → 3'
pMV306-hsp60 HA (Right)	TACCCATACGATGTTCCAGATTACG
Inf P766 (R2)	CATGCGGTTGTGAGCGCTCAC
4c5GP_mmpl10_f	CAATTGTGAGCGCTCACAACCGCATGCGGCGGCTAGCCGATC
HA_mmpl10_r	CTGCAGCTAAGCGTAATCTGGAACATCGTATGGGTAACCTGCCGGCGCGGCGG C
Mmpl10-720down_f	ACCAATGCGGCGGACCCG
L10-720down_L10478up_r	GGGACGGGTCCGCCGCATTGGTGGTGGCCCTGAACTCGGG
QC_MAB0937c_D891AY892F_f	GGTCGGTGCCGCCTTCAACATGCTCTTCGTGTCA
QC_MAB0937c_Y892FD891A_r	TGACACGAAGAGCATGTTGAAGGCGGCACCGACC
Pmv306_f	CGTACGATCGACTGCCAGGC
Linker_r	CGAGCCACCACCGCCCG
Linker_Neongreen_f	GAGTCGGGCGGTGGTGGCTCGATGGTGAGCAAGGGCGAGG
Pmv306_Neongreen_r	GATGCCTGGCAGTCGATCGTACGTCACTTGTACAGCTCGTCCATGC
Tetoff_MAB0936c_f	CCGCAATTGTGAGCGCTCACAACCGCATGAGAAACCTCCTCGCAGGAACAG
HA_MAB0936c_r	GCTAAGCGTAATCTGGAACATCGTATGGGTAATTGGGAGGGGTCGGGCG
MAB-0937c U PacI (F)	GAGATTAATTAACACCTTTGGAATCATCCAGAGCGAG
MAB-0937c U MfeI (R)	GAGACAATTGCCGCCGCATAAATTCCTCACCC
MAB-0937c D MfeI (F)	GAGACAATTGGCAGGTTAGCAAGCACTCGTTATGTATG
MAB-0937c D NheI (R)	GAGAGCTAGCTCGAGCCGATTCAGCGTTTCCTC
MAB-0936c U PacI (F)	GAGATTAATTAAGCGTAGATCAGCTCGTCG
MAB-0937c U MfeI (R)	GAGACAATTGGGAGCACGGATGAGAAAC
MAB-0937c D MfeI (F)	GAGACAATTGCCCAATTAAGCATGACTTGGAC
MAB-0937c D NheI (R)	GAGAGCTAGCGCGAACCGGTCGACGAAG
NheI-mScarlett-For-pMV306	ATAGCTAGCATCTTAATTAAGGCGCCTCATGTTC
XbaI-mScarlett-Rev-pMV306	AGGTCTAGAGATCACCGCGGCCAT

REFERENCES

1. Bernut A, Viljoen A, Dupont C, Sapriel G, Blaise M, Bouchier C, Brosch R, De Chastellier C, Herrmann J-L, Kremer L. 2016. Insights into the smooth-to-rough transitioning in *Mycobacterium bolletii* unravels a functional Tyr residue conserved in all mycobacterial MmpL family members: MmpL activity is dependent on a crucial tyrosine. Mol Microbiol 99:866–883.
2. Aguilera-Correa JJ, Boudehen Y-M, Kremer L. 2023. Characterization of *Mycobacterium abscessus* colony-biofilms based on bi-dimensional images. Antimicrob Agents Chemother e00402-23.
3. Jankute M, Nataraj V, Lee OY-C, Wu HHT, Ridell M, Garton NJ, Barer MR, Minnikin DE, Bhatt A, Besra GS. 2017. The role of hydrophobicity in tuberculosis evolution and pathogenicity. Sci Rep 7:1315.
4. Fröberg G, Maurer FP, Chryssanthou E, Fernström L, Benmansour H, Boarbi S, Mengshoel AT, Keller PM, Viveiros M, Machado D, Fitzgibbon MM, Mok S, Werngren J, Cirillo DM, Alcaide F, Hyyryläinen H-L, Aubry A, Andres S, Nadarajan D, Svensson E, Turnidge J, Giske CG, Kahlmeter G, Cambau E, Van Ingen J, Schön T. 2023. Towards clinical breakpoints for non-tuberculous

mycobacteria – Determination of epidemiological cut off values for the *Mycobacterium avium* complex and *Mycobacterium abscessus* using broth microdilution. Clin Microbiol Infect 29:758–764.

5. Kremer L, Maughan WN, Wilson RA, Dover LG, Besra GS. 2002. The *M. tuberculosis* antigen 85 complex and mycolyltransferase activity. Lett Appl Microbiol 34:233–237.
6. Lamason RL, Mohideen M-APK, Mest JR, Wong AC, Norton HL, Aros MC, Jurynec MJ, Mao X, Humphreville VR, Humbert JE, Sinha S, Moore JL, Jagadeeswaran P, Zhao W, Ning G, Makalowska I, McKeigue PM, O'donnell D, Kittles R, Parra EJ, Mangini NJ, Grunwald DJ, Shriver MD, Canfield VA, Cheng KC. 2005. SLC24A5, a putative cation exchanger, affects pigmentation in zebrafish and humans. Science 310:1782–1786.
7. Ragunathan P, Sae-Lao P, Hamela C, Alcaraz M, Krah A, Poh WH, Ern Pee CJ, Hou Lim AY, Rice SA, Pethe K, Bond PJ, Dick T, Kremer L, Bates RW, Grüber G. 2024. High efficacy of the F-ATP synthase inhibitor TBAJ-5307 against non-tuberculous mycobacteria *in vitro* and *in vivo*. J Biol Chem 300:105618.
8. Wetzel KS, Illouz M, Abad L, Aull HG, Russell DA, Garlena RA, Cristinziano M, Malmsheimer S, Chalut C, Hatfull GF, Kremer L. 2023. Therapeutically useful mycobacteriophages BPs and Muddy require trehalose polyphleates. Nat Microbiol 8:1717–1731.
9. Hatakeyama S, Ohama Y, Okazaki M, Nukui Y, Moriya K. 2017. Antimicrobial susceptibility testing of rapidly growing mycobacteria isolated in Japan. BMC Infect Dis 17:197.
10. Pichler V, Dalkilic, L., Shoaib, T., Shapira, T., Rankine-Wilson, L., Boudehen Y-M, Chao, J., Sexton, D., Prieto, M., Quon, B., Tocheva, E., Kremer, L., Hsiao, W., Av-Gay, Y. The diversity of clinical *Mycobacterium abscessus* isolates in morphology, glycopeptidolipids and infection rates in a macrophage model. J Med Microbiol 73(8).
11. Boudehen Y-M, Tasrini Y, Aguilera-Correa JJ, Alcaraz M, Kremer L. 2023. Silencing essential gene expression in *Mycobacterium abscessus* during infection. Microbiol Spectr 11:e02836-23.
12. Daher W, Leclercq L-D, Johansen MD, Hamela C, Karam J, Trivelli X, Nigou J, Guérardel Y, Kremer L. 2022. Glycopeptidolipid glycosylation controls surface properties and pathogenicity in *Mycobacterium abscessus*. Cell Chem Biol 29:910–924.e7.

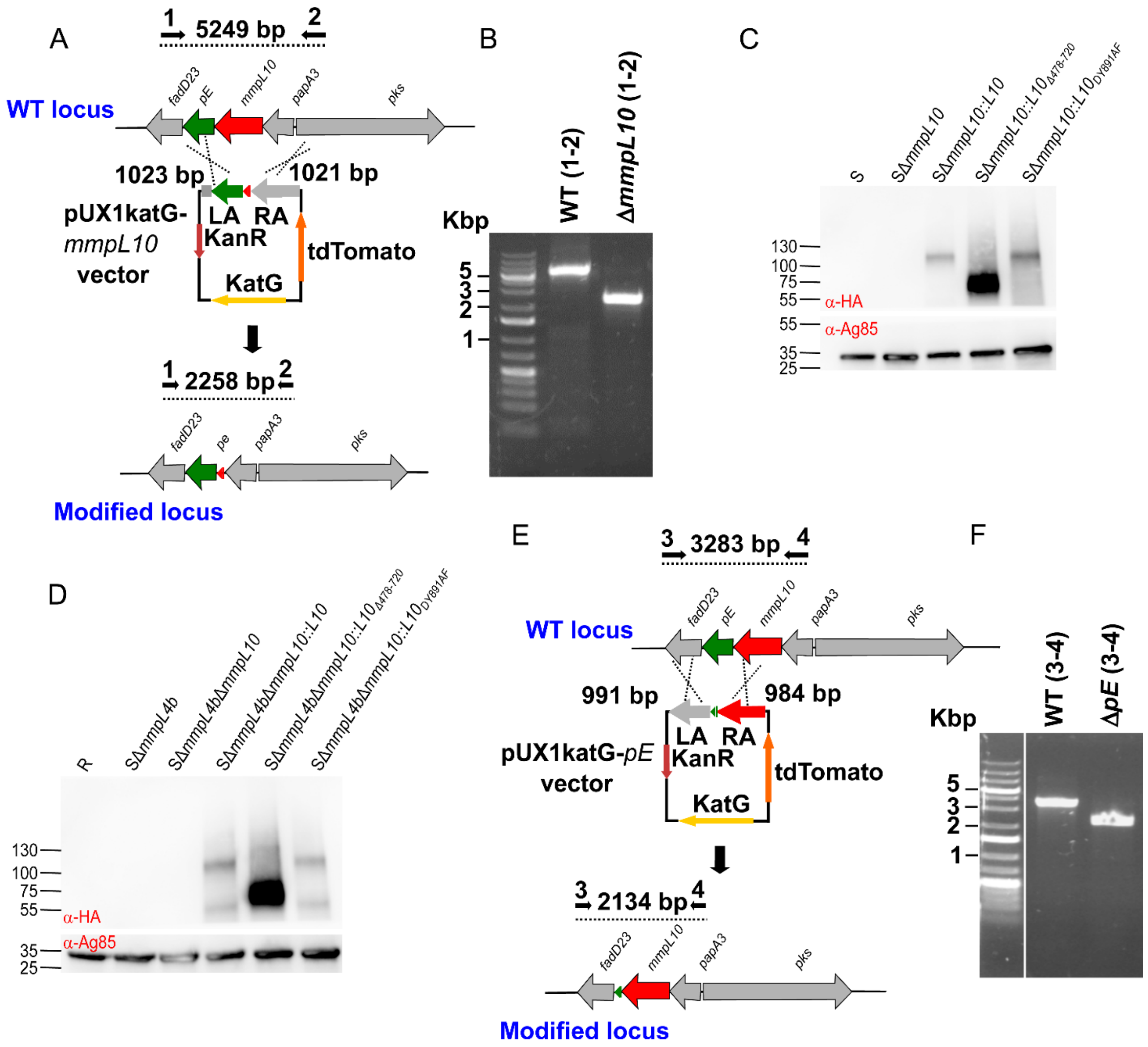

Supplemental Figure 1. Homologous recombination strategy used to generate Δ*mmpL10* and Δ*pE* mutants **(A and E)**. Confirmation of *mmpL10* and *pE* deletions in *M. abscessus* by PCR **(B and F)**. Immunoblot analysis of full-length MmpL10, $MmpL10_{\Delta 478\text{-}720}$, and $MmpL10_{DY891AF}$ expression in *M. abscessus* S Δ*mmpL10* and S Δ*mmpL4b*/Δ*mmpL10* strains, using anti-HA antibodies **(C and D, upper panels)**. The Ag85 protein complex served as a loading control **(C and D, lower panels)**.

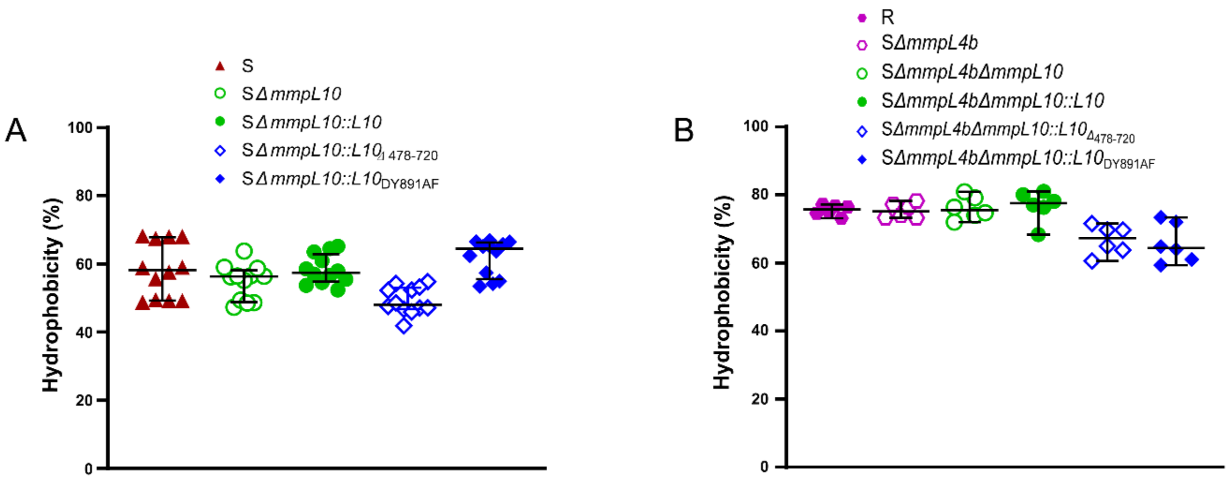

Supplemental Figure 2. The knock-out of *mmpL10* does not modify hydrophobicity in the S **(A)** or R **(B)** morphotypes. The hydrophobicity experiment was performed using three biological replicates with three technical replicates per biological replicate. The error bar denotes the interquartile range.

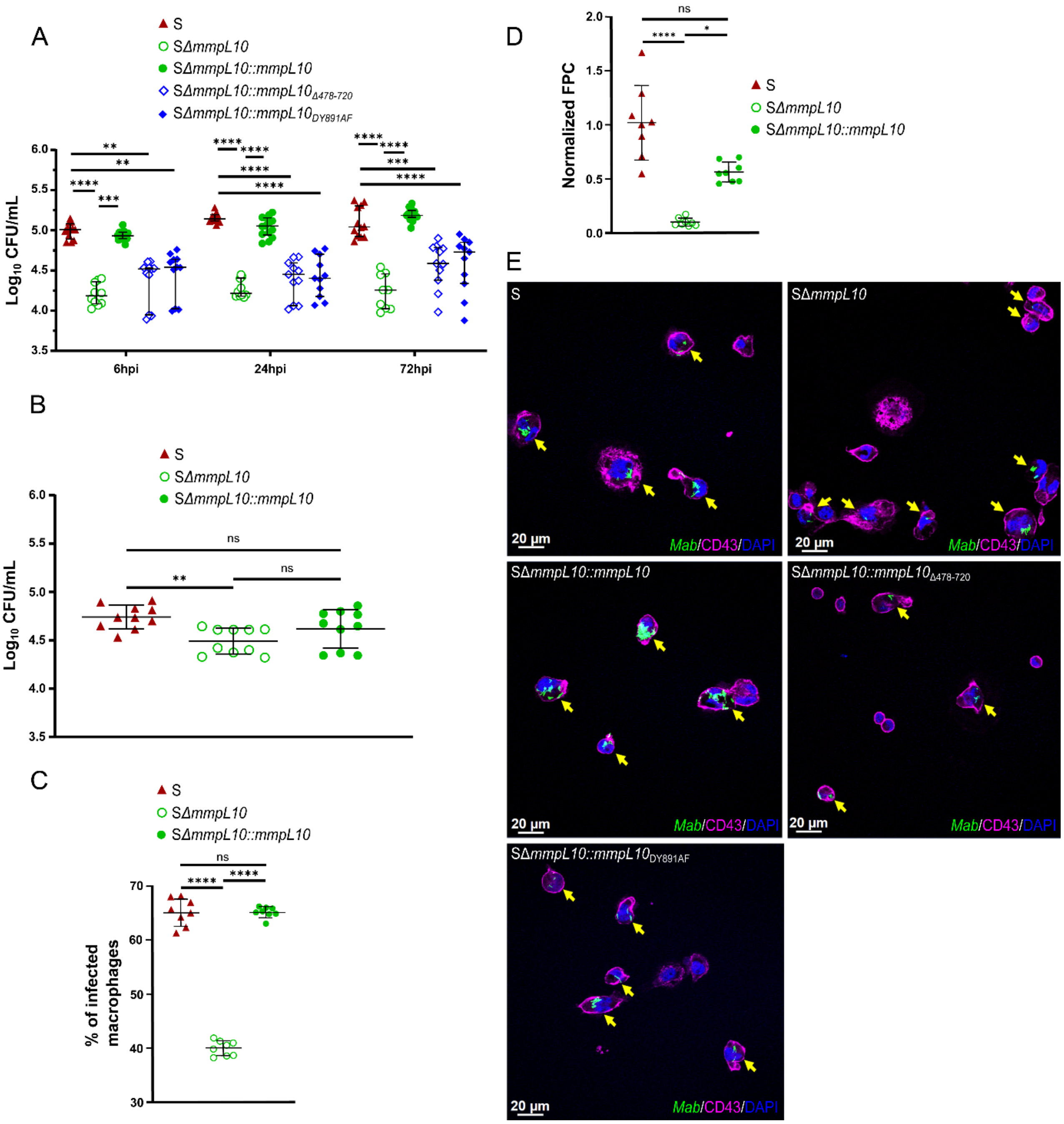

Supplemental Figure 3. (A) CFUs of an *mmpL10* deletion mutant of *M. abscessus* S, the parent strain and strains complemented with the full-length (*mmpL10*), *mmpL10*$_{\Delta478\text{-}720}$ and *mmpL10*$_{DY891AF}$ genes were determined at 6, 24, and 72 hpi. Data are median ± interquartile range for at least 3 independent experiments (each time in quadruplicate). Statistical analysis was performed using Kruskal-Wallis test with Dunn's multiple comparisons test. **(B)** Attachment of *mmpL10*-deficient strains is slightly reduced compared to the parental strain. CFUs were determined 30 min post-infection after incubation on ice. Data are median ± interquartile range for at least 3 independent experiments (each time in quadruplicate). Statistical analysis was performed using ordinary one-way ANOVA with Šídák's multiple comparisons test. **(C)** Percentage of infected THP-1 macrophages at 72 hpi. Data are shown as individual technical replicates within at least three independent experiments, with the median and interquartile range. Statistical analysis was performed using Kruskal-Wallis test with Dunn's multiple comparisons test. **(D)** Bacterial load quantified by fluorescent pixel counts (FPC). Data were normalized to the S WT and are shown as individual technical replicates within at

least three independent experiments, with the median and interquartile range. Statistical analysis was performed using Kruskal-Wallis test with Dunn's multiple comparisons test. **(E)** Five immunofluorescent fields were taken at 72 hpi using a confocal microscope (40x magnification), showing the macrophages infected with the various strains (in green). The nuclei are shown in dark blue, the CD43 protein associated with the plasma membrane of macrophages is in magenta. Bacilli are inside the infected macrophages. ns: non-significant, *$p<0.05$, **$p<0.01$, ***$p<0.001$, ****$p<0.0001$.

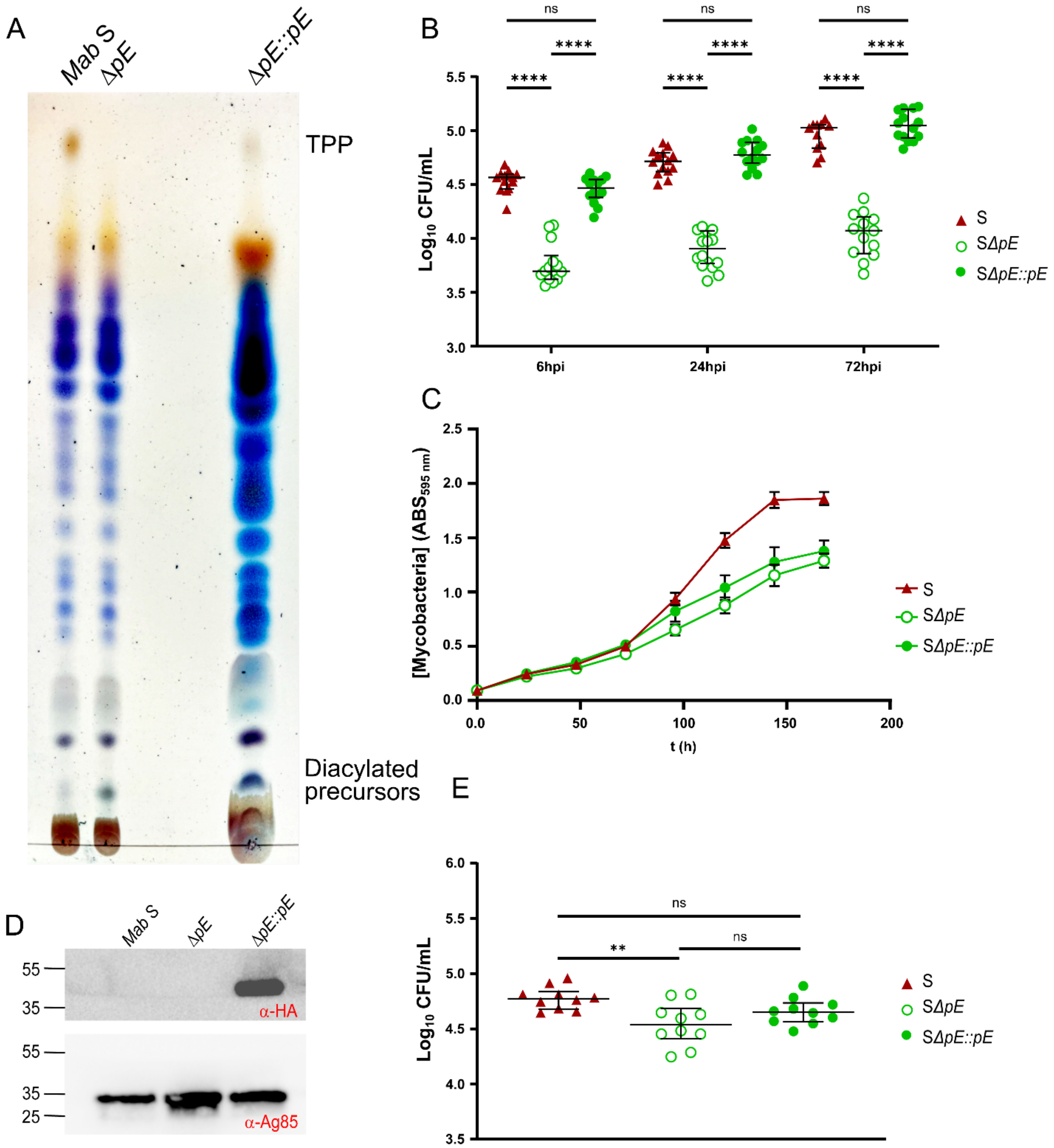

Supplemental Figure 4. (A) TLC analysis of total lipids extracted from a *pE* deletion mutant of *M. abscessus*, its parent (S) and complemented strain. Eluent: $CHCl_3/CH_3OH$ (90:10, v/v). TLC was revealed by spraying 0.2% anthrone on the plate, followed by charring. **(B)** CFUs of a *pE* deletion mutant of *M. abscessus* S, the parent and complemented strains were determined at 6, 24, and 72 hpi. Data are median ± interquartile range for at least three independent experiments (each time in quadruplicate). **(C)** Growth of the S, Δ*pE* and Δ*pE::pE* strains in liquid medium. The experiment was performed using two biological replicates with six technical replicates per biological replicate. **(D)** Overexpression of pE-HA in *M. abscessus* S Δ*pE*. Immunoblotting of pE expression in the complemented strain using anti-HA antibodies (upper panel). The Ag85 protein complex was used as a loading control (lower panel). **(E)** Attachment of Δ*pE* is slightly reduced compared to the parental S strain. CFUs were determined 30 min post-infection after incubation on ice. Data are median ± interquartile range for at least three independent experiments (each time in quadruplicate). Statistical analysis was performed using ordinary one-way ANOVA with Šídák's multiple comparisons test. ns: non-significant, ***p*<0.01; ****p*<0.0001.

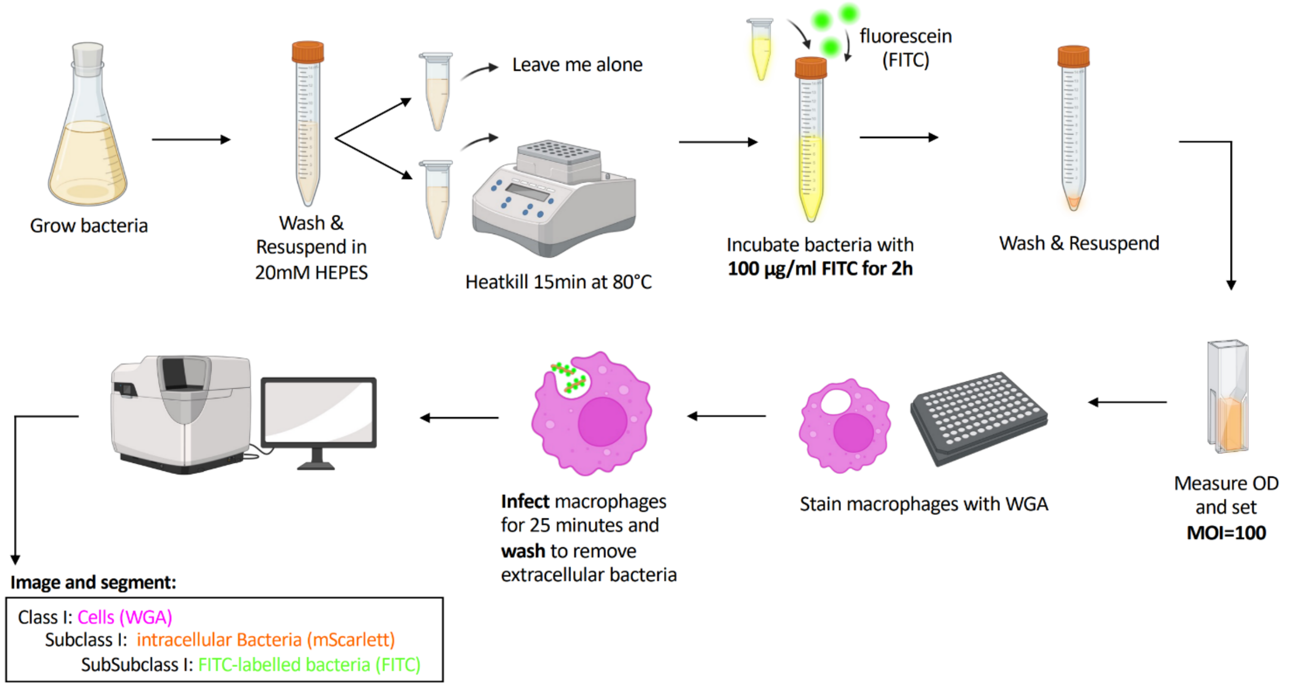

Supplemental Figure 5. Analysis of phagosomal acidification using fluorescein (FITC). Red fluorescent bacteria are labelled with the green, pH-dependent fluorophore FITC. After infection of WGA-stained THP-1 macrophages with an MOI of 100 for 25 min, the evolution of the FITC intensity (Sum.intensity.pixel.FITC) per bacterium (Area.mScarlett) was measured and analyzed every 20 min by confocal microscopy.

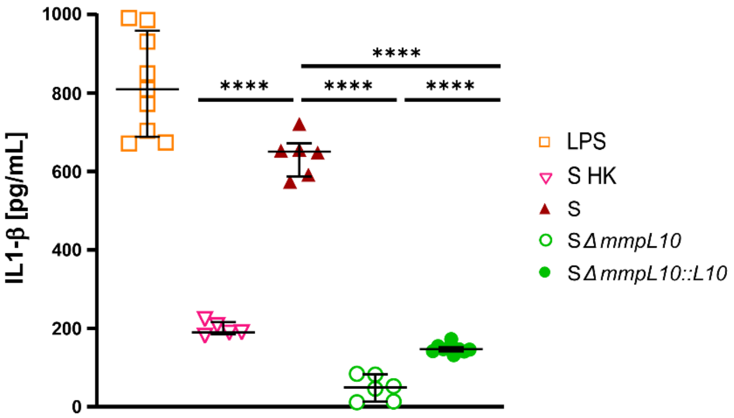

Supplemental Figure 6. The response to infection with an *mmpL10* deletion mutant of *M. abscessus* S, the parent and complemented strain was assessed by quantifying IL-1β secretion. HK, heat-killed *M. abscessus* S. Histograms with error bars represent median ± interquartile range for at least 3 independent experiments. LPS was included as a positive control. Statistical analysis was performed using Kruskal-Wallis test with Dunn's multiple comparisons test. ****p<0.0001.

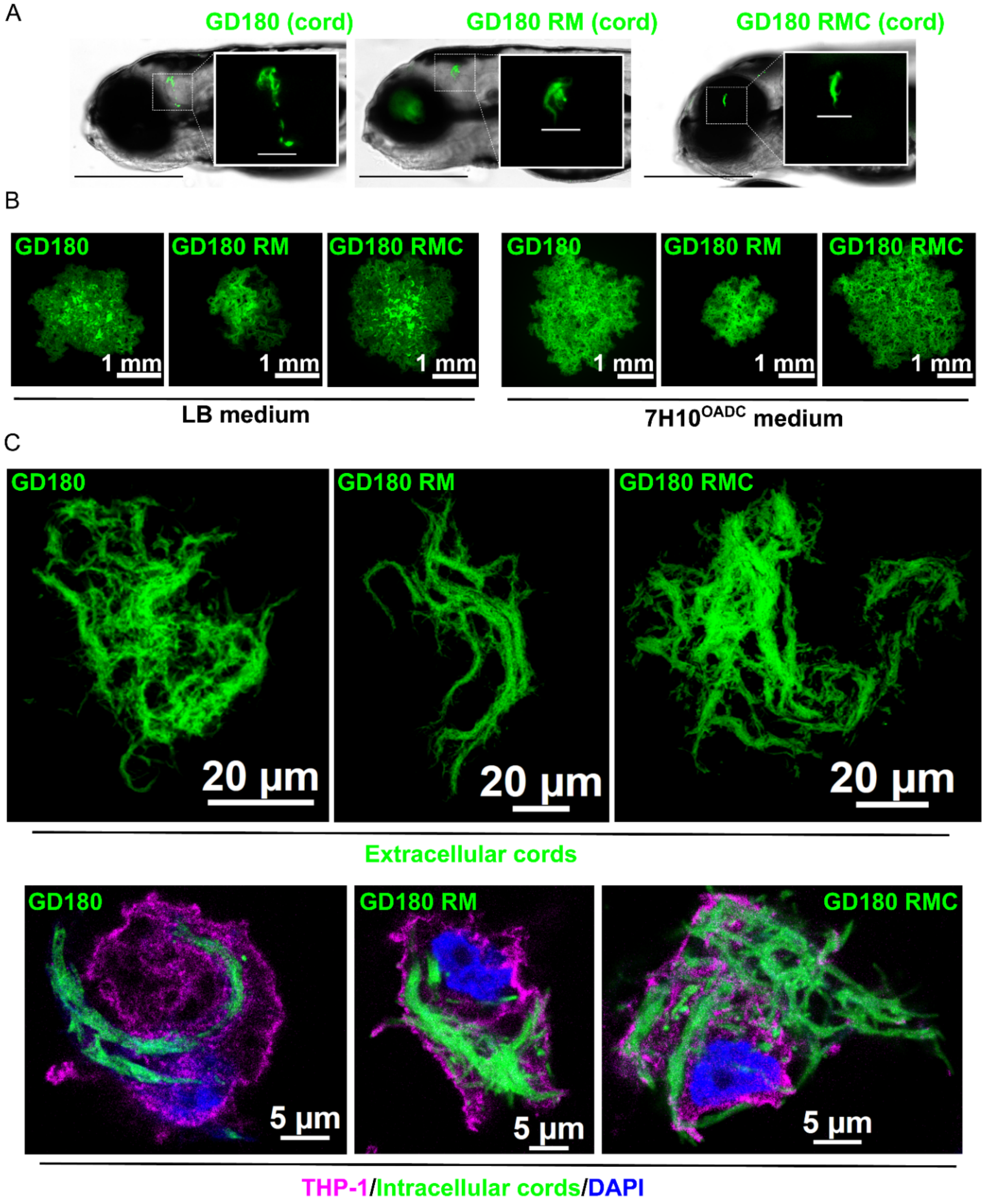

Supplemental Figure 7. Disruption of TPP in GD180 RM does not impact on cord formation in zebrafish **(A)**, on agar plates **(B)** or following infection of macrophages **(C)**. **(A)** Cording of GD180, GD180 RM and GD180 RMC in zebrafish embryos. The green overlay represents mWasabi-expressing strains at 3 dpi (black scale bar: 500 µm). Larvae infected with all three strains developed serpentine cords, as shown in the enlarged fluorescent views (white scale bar: 70 µm). **(B)** The different strains expressing mWasabi (green) were cultivated on LB or $7H10^{OADC}$ agar plates. **(C)** Single extracellular cords growing outside of the macrophage were imaged at 3 dpi **(upper panel)**. Representative infected macrophages captured at 3 dpi and containing intracellular cords **(lower panel)**. The macrophage surface was visualized using anti-CD43 antibodies (magenta), and the nuclei were stained with DAPI (blue). All strains examined produced both intracellular and extracellular cords.
